# Prioritizing Health Care Strategies to Reduce Childhood Mortality

**DOI:** 10.1001/jamanetworkopen.2022.37689

**Published:** 2022-10-21

**Authors:** Zachary J. Madewell, Cynthia G. Whitney, Sithembiso Velaphi, Portia Mutevedzi, Sana Mahtab, Shabir A. Madhi, Ashleigh Fritz, Alim Swaray-Deen, Tom Sesay, Ikechukwu U. Ogbuanu, Margaret T. Mannah, Elisio G. Xerinda, Antonio Sitoe, Inacio Mandomando, Quique Bassat, Sara Ajanovic, Milagritos D. Tapia, Samba O. Sow, Ashka Mehta, Karen L. Kotloff, Adama M. Keita, Beth A. Tippett Barr, Dickens Onyango, Elizabeth Oele, Kitiezo Aggrey Igunza, Janet Agaya, Victor Akelo, J. Anthony G. Scott, Lola Madrid, Yunus-Edris Kelil, Tadesse Dufera, Nega Assefa, Emily S. Gurley, Shams El Arifeen, Ellen A. Spotts Whitney, Katherine Seib, Chris A. Rees, Dianna M. Blau

**Affiliations:** 1 Center for Global Health, US Centers for Disease Control and Prevention, Atlanta, Georgia; 2 Emory Global Health Institute, Emory University, Atlanta, Georgia; 3 Chris Hani Baragwanath Academic Hospital, School of Clinical Medicine, Faculty of Health Sciences, University of the Witwatersrand, Johannesburg, South Africa; 4 South African Medical Research Council Vaccines and Infectious Diseases Analytics Research Unit, University of the Witwatersrand, Johannesburg, South Africa; 5 Department of Obstetrics and Gynaecology, University of Ghana Medical School, Accra, Ghana; 6 Ministry of Health and Sanitation, Freetown, Sierra Leone; 7 Crown Agents, Freetown, Sierra Leone; 8 Centro de Investigação em Saúde de Manhiça, Maputo, Mozambique; 9 Instituto Nacional de Saúde, Maputo, Mozambique; 10 ISGlobal–Hospital Clínic, Unversitat de Barcelona, Barcelona, Spain; 11 Institutó Catalana de Recerca I Estudis Avançats, Barcelona, Spain; 12 Pediatrics Department, Hospital Sant Joan de Déu, Universitat de Barcelona, Esplugues, Barcelona, Spain; 13 Consorcio de Investigación Biomédica en Red de Epidemiología y Salud Pública, Madrid, Spain; 14 Department of Pediatrics and Department of Medicine, Center for Vaccine Development and Global Health, University of Maryland School of Medicine, Baltimore; 15 Centre pour le Développement des Vaccins, Ministère de la Santé, Bamako, Mali; 16 Centers for Disease Control and Prevention–Kenya, Kisumu, Kenya; 17 Kisumu County Department of Health, Kisumu, Kenya; 18 Kenya Medical Research Institute-Center for Global Health Research, Kisumu, Kenya; 19 Department of Infectious Disease Epidemiology, London School of Hygiene and Tropical Medicine, London, United Kingdom; 20 College of Health and Medical Sciences, Haramaya University, Harar, Ethiopia; 21 International Center for Diarrhoeal Diseases Research, Dhaka, Bangladesh; 22 Department of Epidemiology, Johns Hopkins Bloomberg School of Public Health, Baltimore, Maryland; 23 International Association of National Public Health Institutes, Global Health Institute, Emory University, Atlanta, Georgia; 24 Division of Pediatric Emergency Medicine, Emory University School of Medicine, Children's Healthcare of Atlanta, Atlanta, Georgia

## Abstract

**Importance:**

Although child mortality trends have decreased worldwide, deaths among children younger than 5 years of age remain high and disproportionately circumscribed to sub-Saharan Africa and Southern Asia. Tailored and innovative approaches are needed to increase access, coverage, and quality of child health care services to reduce mortality, but an understanding of health system deficiencies that may have the greatest impact on mortality among children younger than 5 years is lacking.

**Objective:**

To investigate which health care and public health improvements could have prevented the most stillbirths and deaths in children younger than 5 years using data from the Child Health and Mortality Prevention Surveillance (CHAMPS) network.

**Design, Setting, and Participants:**

This cross-sectional study used longitudinal, population-based, and mortality surveillance data collected by CHAMPS to understand preventable causes of death. Overall, 3390 eligible deaths across all 7 CHAMPS sites (Bangladesh, Ethiopia, Kenya, Mali, Mozambique, Sierra Leone, and South Africa) between December 9, 2016, and December 31, 2021 (1190 stillbirths, 1340 neonatal deaths, 860 infant and child deaths), were included. Deaths were investigated using minimally invasive tissue sampling (MITS), a postmortem approach using biopsy needles for sampling key organs and fluids.

**Main Outcomes and Measures:**

For each death, an expert multidisciplinary panel reviewed case data to determine the plausible pathway and causes of death. If the death was deemed preventable, the panel identified which of 10 predetermined health system gaps could have prevented the death. The health system improvements that could have prevented the most deaths were evaluated for each age group: stillbirths, neonatal deaths (aged <28 days), and infant and child deaths (aged 1 month to <5 years).

**Results:**

Of 3390 deaths, 1505 (44.4%) were female and 1880 (55.5%) were male; sex was not recorded for 5 deaths. Of all deaths, 3045 (89.8%) occurred in a healthcare facility and 344 (11.9%) in the community. Overall, 2607 (76.9%) were deemed potentially preventable: 883 of 1190 stillbirths (74.2%), 1010 of 1340 neonatal deaths (75.4%), and 714 of 860 infant and child deaths (83.0%). Recommended measures to prevent deaths were improvements in antenatal and obstetric care (recommended for 588 of 1190 stillbirths [49.4%], 496 of 1340 neonatal deaths [37.0%]), clinical management and quality of care (stillbirths, 280 [23.5%]; neonates, 498 [37.2%]; infants and children, 393 of 860 [45.7%]), health-seeking behavior (infants and children, 237 [27.6%]), and health education (infants and children, 262 [30.5%]).

**Conclusions and Relevance:**

In this cross-sectional study, interventions prioritizing antenatal, intrapartum, and postnatal care could have prevented the most deaths among children younger than 5 years because 75% of deaths among children younger than 5 were stillbirths and neonatal deaths. Measures to reduce mortality in this population should prioritize improving existing systems, such as better access to antenatal care, implementation of standardized clinical protocols, and public education campaigns.

## Key Points

**Question:**

Which public health care improvements could have prevented the most deaths among children younger than 5 years?

**Findings:**

In this cross-sectional study of 3390 deaths investigated at sites with high child mortality rates in sub-Saharan Africa and Southern Asia, 77% were deemed potentially preventable. Recommended measures to prevent deaths were improvements in antenatal and obstetric care, clinical management and quality of care, health-seeking behavior, and health education.

**Meaning:**

These findings suggest that investments in interventions that reduce child mortality should focus on health system improvement, including improved antenatal care and higher quality of pediatric care.

## Teaser

This cross-sectional study investigates which health care and public health improvements could have prevented the most stillbirths and deaths in children younger than 5 years using data from the Child Health and Mortality Prevention Surveillance (CHAMPS) network.

## Introduction

The global mortality rate among children younger than 5 years decreased by 59% from 1990 to 2019.^1^ However, high mortality among children younger than 5 years persists across regions and countries, with sub-Saharan Africa and Southern Asia accounting for 81% of the 5.0 (95% CI, 4.8-5.5) million deaths among children younger than 5 years in 2020.^2^ The United Nations adopted the Sustainable Development Goals (SDGs) in 2015 to promote healthy lives for children and eliminate preventable deaths among newborns and children younger than 5 years by 2030.^3^ SDG 3.2.1 specifically aims to reduce newborn mortality to fewer than 12 per 1000 live births and mortality among children younger than 5 years to fewer than 25 per 1000 live births in every country.^3^ Furthermore, the World Health Organization (WHO) and United Nations Children’s Fund launched the Every Newborn Action Plan as a blueprint for improving newborn health and ending preventable stillbirths by 2035.^4^

High mortality among children younger than 5 years in sub-Saharan Africa and Southern Asia has been attributed to suboptimal health care quality,^5^ long distances from home and access challenges to health care facilities,^6,7^ and delays in seeking timely health care.^8^ Interventions that could substantially reduce mortality among children younger than 5 years include better quality of clinical and antenatal care (ANC), access to emergency obstetrical procedures, enhanced triage and risk-stratification, immunization coverage, and infection control measures.^9^ However, a comprehensive analysis of public health and clinical interventions that would produce the greatest reduction of mortality among children younger than 5 years in sub-Saharan Africa and Southern Asia is lacking.

We analyzed data from the Child Health and Mortality Prevention Surveillance (CHAMPS) network, which currently operates in 7 sites in Africa and Southern Asia to track causes of stillbirths and deaths among children younger than 5 years. A core objective of this is to increase child survival, and for this, the assessment of each individual death includes evaluation of its potential preventability and which actions would have been needed to prevent it. This assessment of pathological and modifiable causes of deaths by a multidisciplinary expert review panel could result in broad understanding of health system deficiencies that may inform targeted interventions to decrease stillbirths and childhood mortality.

## Methods

### Data Sources

CHAMPS collects standardized, population-based, surveillance data from sites with high child mortality to understand and track preventable causes of death. CHAMPS currently includes sites in 7 countries: Bangladesh, Ethiopia, Kenya, Mali, Mozambique, Sierra Leone, and South Africa. CHAMPS is not intended to be representative of entire countries but rather focuses on regions where mortality rates are known to be highest. To that end, understanding causes of death (and what it would take to prevent them) at these sites could have the greatest impact in terms of reducing mortality. By design, CHAMPS does not reflect low mortality areas. The CHAMPS database contains comprehensive data on all stillbirths and deaths among children younger than 5 years enrolled at each of the surveillance sites. These data include demographic characteristics, extensive postmortem diagnostic results, clinical medical record abstraction data for each child and, when appropriate, maternal antenatal records and verbal autopsy data (as well as social autopsy data in Sierra Leone). Site characteristics, selection criteria, catchment areas, death notification methods, eligibility screening, and specimen and data collection methods have been previously described.^10,11^ Limitations of the CHAMPS methodology have been documented elsewhere^10,12,13^ and include the inability to include all deaths within catchment areas, disparate population characteristics between sites, and overrepresentation of health care facility–based deaths. Ethical approval was obtained for use of CHAMPS data by each site’s ethical review board and by the Emory University Rollins School of Public Health. Parents or guardians of stillborn fetuses or deceased children provided written informed consent before collection of data, specimens, or information on the mothers. All cases were anonymized prior to review. The Strengthening the Reporting of Observational Studies in Epidemiology (STROBE) reporting guidelines for cross-sectional studies were followed.

### Causes of Death

Details regarding the cause of death determination and standardization across sites processes have been described elsewhere.^11,12^ Briefly, deaths are investigated with minimally invasive tissue sampling (MITS), a postmortem approach using biopsy needles for sampling key organs and body fluids. The samples undergo testing using conventional microbiology and multiplexed polymerase chain reaction (PCR) assays using TaqMan array cards; tissues are also examined by pathologists and subject to more advanced histopathological tests. Any available data regarding the terminal events are abstracted from medical records and verbal autopsy and recorded from caregiver recollection. A determination of cause of death (DECODE) panel consisting of pediatricians, obstetricians, epidemiologists, pathologists, microbiologists, and other health care professionals review case data at each surveillance site to assign causes of death. CHAMPS uses the WHO *International Statistical Classification of Diseases and Related Health Problems, Tenth Revision *(*ICD-10*) and the WHO application of *ICD-10* deaths during the perinatal period (*ICD-PM*).^14,15^ For deaths in which only a single cause led to death, that cause is listed as the underlying cause. For deaths in which multiple causes led to the death, the panel determines the causal chain including the underlying, antecedent, and immediate causes leading to death.^13^ The underlying cause usually occurred before immediate or antecedent conditions and may have predisposed the child to an immediate cause or comorbid illnesses that then led to death; the immediate cause was closest to the death, and the antecedent causes were in between the underlying and immediate causes. Each death has only 1 underlying cause, zero or 1 immediate cause, and zero or more antecedent causes. At the site level, a subset of cases that underwent DECODE review are shared with the other sites for secondary review as a quality control measure.

### Health System Improvements

For each death, the DECODE panel determined whether the death was preventable (yes, no, or under certain circumstances) by considering all the information available for each case, which may include demographic, clinical, pathological, microbiological, verbal autopsy, photography, and anthropometric measurements. The definition of preventability mainly captures the conditions immediately surrounding the death of that particular child and not the broader global political, financial, and social influences. If the death was deemed potentially preventable, the panel identified predetermined health system gaps (Table) and recommended improvements based on those gaps that could have prevented the death. These 10 categories emerged from categorization of the free text responses derived from the first DECODE panel evaluations from 2016 to 2017. These categories are still evolving. Each death could have multiple prevention categories listed. For each preventable death, the panel also had the option to provide specific public health action recommendations beyond the 10 categories in an open text field, which were subsequently categorized as well.

**Table.** Health System Improvements and Examples of Specific Public Health Actions Recommended by the Expert Determination of Cause of Death Panel

Health system improvement category	Public health action
Improved clinical management and quality of care	Advanced respiratory support, improvements in medical records, properly trained staff for parturition and health care
Improved antenatal and obstetric care and management	Ultrasonography, timely caesarian delivery, management of preeclampsia
Improved health-seeking behavior	Regular antenatal check-ups, early recognition of illness, and early referral for treatment at a health care facility
Improved infection prevention and control	Personal hygiene, environmental sanitation, appropriate use of antibiotics
Improved health education	Immunizations, preventing malnutrition, bed nets to prevent malaria
Improved nutritional support	Management of malnutrition
Improved HIV prevention and control	Maternal access to testing and antiretroviral therapy, therapy for neonatal infections
Improved family planning	Prevention of unwanted pregnancies
Improved use of existing vaccinations	Pneumococcal conjugate vaccine, *Haemophilus influenzae* type b vaccine
Improved transport system	Road infrastructure, availability of public transportation, availability of resources (eg, oxygen) on ambulances

There were 10 high-level categories of health system improvements (Table), and each death could have multiple prevention categories listed. Although some health system improvement categories primarily target specific age groups (eg, improvements in antenatal and obstetric care), any category implemented at a given site may affect children in other age groups to varying degrees. We evaluated health system improvement categories across all sites by age group. We defined stillbirths as the death of a baby before or at delivery, neonates as those aged 0 to 27 days, and infants and children as those aged 28 days to younger than 5 years.^16^

### Statistical Analysis

To determine which health system improvements could have prevented the most deaths regardless of cause, we generated every combination of 1 to 10 categories (1023 combinations) and calculated how many deaths could have been prevented for each combination under the assumption (A1) that all health system improvement categories recommended for a single death are necessary to prevent that death. We also conducted sensitivity analyses assuming (A2) deaths would be reduced proportionally to the number of categories implemented for deaths with multiple health system improvement categories noted, and (A3) any single category among categories recommended for each death is sufficient to prevent the death. For example, if 4 health system improvement categories were recommended for a set of deaths and only 1 was implemented, we calculated that, according to the 3 assumptions: (A1) those deaths would not be prevented, (A2) 25% of those deaths would be prevented, and (A3) 100% of those deaths would be prevented (eMethods in Supplement 1). All analyses were done in R version 4.1.2 (R Foundation for Statistical Computing).

## Results

Between December 2016 and December 2021, there were 9354 CHAMPS-eligible stillbirths and deaths among children younger than 5 years, of which 4331 (46.3%) were enrolled in CHAMPS and consented for MITS (eFigure 1 in Supplement 1). Our study included 3390 deaths with MITS performed that were also reviewed and coded by DECODE panels: 1190 stillbirths, 1340 neonates, and 860 infants and children younger than 5 years (eTable 1 in Supplement 1). Of all deaths, 3045 (89.8%) occurred in a health care facility and 344 (11.9%) in the community. There were 1505 (44.4%) female deaths and 1880 (55.5%) male deaths; sex was not recorded for 5 deaths. There were 832 deaths from South Africa, 654 from Mozambique, 578 from Kenya, 420 from Sierra Leone, 364 from Bangladesh, 334 from Ethiopia, and 208 from Mali.

The most frequent causes of death anywhere in the causal pathway across all sites were intrapartum asphyxia or hypoxia for stillbirths (952 [80.0%]); for neonatal deaths, preterm birth complications (588 [43.9%]), intrapartum asphyxia or hypoxia (487 [36.3%]), and sepsis (484 [36.1%]); and for infant and child deaths, pneumonia (373 [43.4%]), sepsis (346 [40.2%]), malnutrition (211 [24.5%]), and malaria (180 [20.9%]) (eTable 2 in Supplement 1). Deaths may have multiple causes listed, so causes of death for each age group may exceed 100%. Nearly half of the deaths (1579 of 3390 [46.5%]) resulted from multiple causes.

**Figure 1.**
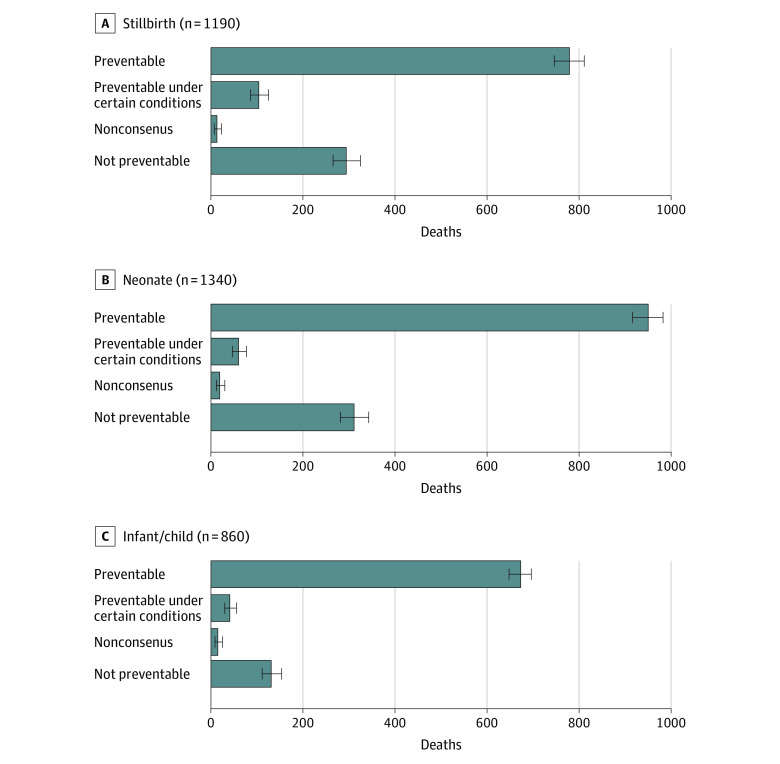
Number of Deaths Deemed Preventable Across All Sites by Age Group, December 2016 to December 2021 A total of 3390 deaths were included. Nonconsensus indicates that the expert panel was unable to agree as to whether the death could have been prevented. Whiskers indicate 95% CIs.

Of 3390 deaths across all sites, 883 stillbirths (74.2%), 1010 neonatal deaths (75.4%), and 714 infant or child deaths (83.0%) were deemed preventable or preventable under certain circumstances by the DECODE panelists (Figure 1). The proportion of all stillbirths, neonatal deaths, and infant or child deaths that were deemed preventable was highest in Kenya (554 of 578 [95.8%]), Ethiopia (320 of 334 [95.8%]), and Bangladesh (344 of 364 [94.5%]) (eTable 3 in Supplement 1). Of the 5 most frequent causes of death anywhere in the causal pathway, deaths from malaria (177 of 180 [98.3%]) were deemed most preventable for infants and children, whereas deaths from perinatal asphyxia or hypoxia (423 of 498 [86.9%]) were deemed most preventable for neonates (eFigure 2 in Supplement 1). There were 736 deaths across all age groups that were deemed unpreventable with asphyxia or hypoxia (239 [32.5%]), sepsis (169 [23.0%]), preterm birth complications (162 [22.0%]), and congenital birth defects (153 [20.8%]) as the most frequent causes.

**Figure 2.**
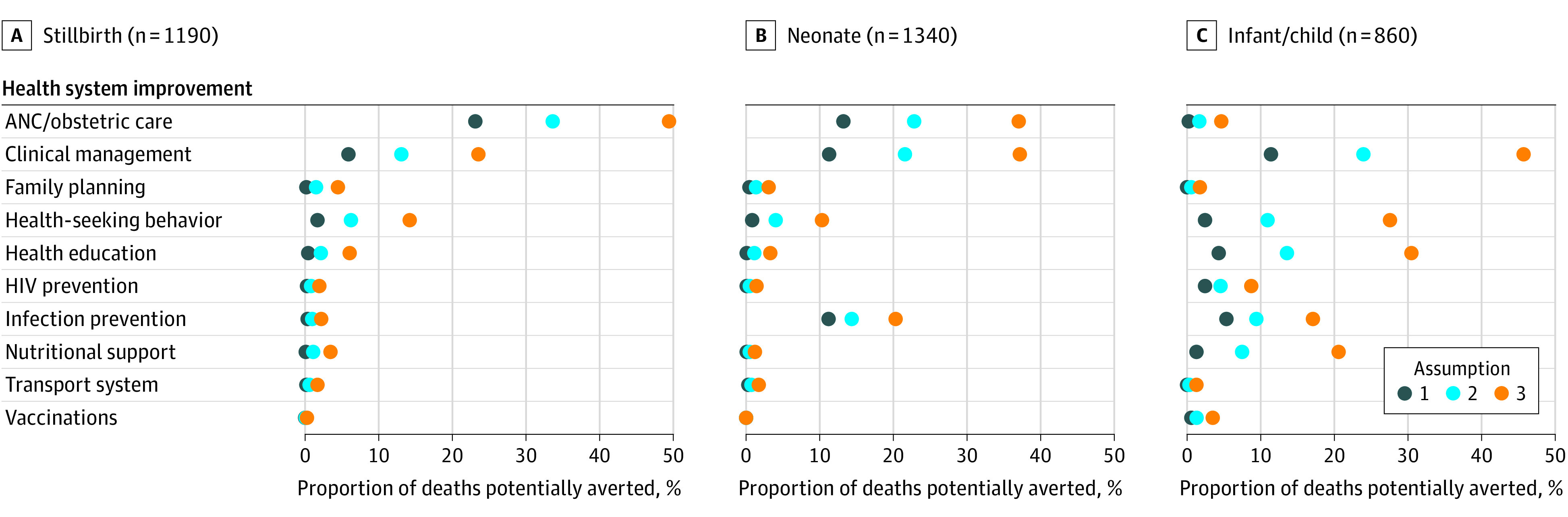
Proportion of 3390 Deaths That Could Have Been Prevented for Each Health System Improvement Category Across All Sites by Age Group, December 2016 to December 2021 This figure assumes (1) all recommendations given for a single death are necessary to prevent that death, (2) for every category implemented for deaths with multiple categories, deaths would be reduced proportionally, and (3) any single category among all categories recommended for each death is sufficient to prevent that death. Categories appear in the Table. ANC indicates antenatal care.

We examined which single health system improvement category could have prevented the most deaths across all sites assuming all improvement categories recommended for a single death are necessary to prevent that death (Figure 2). The health system improvement categories that could have prevented the most deaths were improved antenatal and obstetric care (stillbirths, 275 [23.1%]; neonates, 177 [13.2%]) and improved clinical management (stillbirths, 70 [5.9%]; neonates, 151 [11.3%]; infants and children, 98 [11.4%]) (Table and eTable 4 in Supplement 1). Overall, improved antenatal and obstetric care was recommended in 588 stillbirths (49.4%). These categories could primarily have prevented perinatal asphyxia or hypoxia for stillbirths and neonates, followed by preterm birth complications and sepsis (eFigure 3 in Supplement 1). Improved infection prevention and control (IPC), however, could have prevented the most deaths in South Africa, where nosocomial infections have been frequently documented during CHAMPS (neonates, 141 of 436 [32.3%]; infants and children: 42 of 221 [19.0%]) (eTable 5 in Supplement 1). Improved health-seeking behavior and health education were recommended in 237 (27.6%) and 262 (30.5%) of 860 child and infant deaths, respectively. Repeating the analysis using the assumptions that deaths would be reduced proportionally to the number of health system improvement categories implemented (A2) or that any single category among categories recommended for each death is sufficient to prevent the death (A3) indicated larger proportions of deaths prevented for any single health system improvement category for each age group (Figure 2), such as improved nutritional support, health-seeking behavior, and health education in Ethiopia, Kenya, and Sierra Leone (eTable 5 in Supplement 1). For example, improved clinical management could have prevented 155 stillbirths (13.0%) under A2 and 280 stillbirths (23.5%) under A3, 289 neonatal deaths (21.6%) under A2 and 498 neonatal deaths (37.2%) under A3, and 206 infant or children deaths (24.0%) under A2 and 393 infant or child deaths (45.7%) under A3.

Next, we examined which combinations of the 10 health system improvement categories could have prevented the most deaths (eFigure 4 in Supplement 1). The optimal combination of 5 categories (improved clinical management, infection prevention, health-seeking behavior, health education, and nutritional support) could have prevented 461 infant and child deaths (53.6%) across all sites assuming all categories are necessary (A1) and 823 (72.4%) assuming any single category implemented is sufficient (A3), including 95 (77.9%) and 112 (97.5%) of 122 anemia deaths, 121 (67.2%) and 175 (97.2%) of 180 malaria deaths, and 138 (65.4%) and 188 (89.1%) of 211 malnutrition deaths (eFigure 5 in Supplement 1). The optimal combinations of categories vary by site (eTable 6 in Supplement 1). For the top 5 causes of death across all sites for each age group, we also identified the top combinations of 1 to 3 health system improvement categories that could have prevented the most deaths (eTables 7, 8, and 9 in Supplement 1).

**Figure 3.**
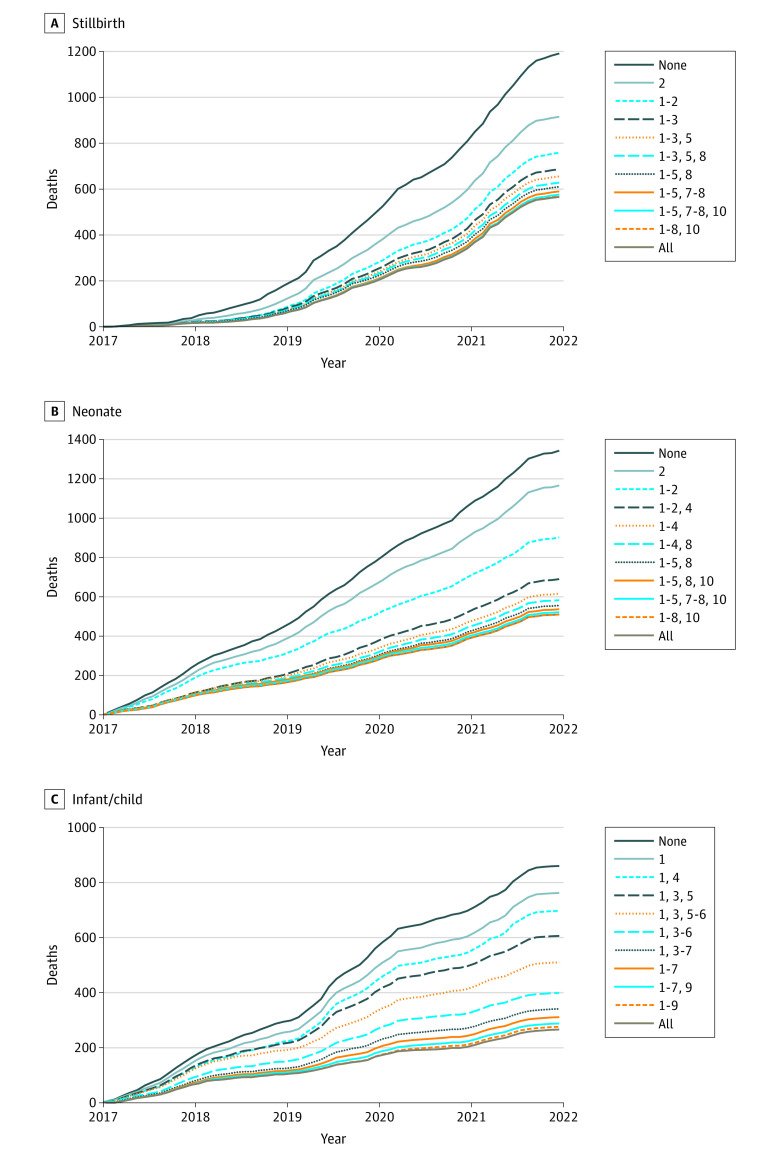
Cumulative Number of Deaths Over Time and Hypothetical Reduction in Deaths From Implementing Optimal Combinations of 1 to 10 Health System Improvement Categories Across All Sites by Age Group, December 2016 to December 2021 This figure assumes all recommendations given for a single death are necessary to prevent that death. None indicates no health system improvements and represents the actual number of deaths over time. 1 indicates improved clinical management and quality of care; 2, improved antenatal and obstetric care and management; 3, improved health-seeking behavior; 4, improved infection prevention and control; 5, improved health education, eg, immunizations, preventing malnutrition, diarrhea, malaria, burns, poisoning; 6, improved nutritional support; 7, improved HIV prevention and control; 8, improved family planning; 9, improved use of existing vaccinations; and 10, improved transport system.

Given that most deaths among children younger than 5 years were among neonates or were stillbirths in our surveillance population, a health system improvement that could have prevented most of the stillbirths and early neonatal deaths (eg, improved antenatal and obstetric care) could have prevented most of the preventable deaths among children younger than 5 years. For example, dual improvements of antenatal and obstetric care and clinical management could have prevented 973 deaths (28.7%) assuming all categories recommended are necessary to prevent that death (Figure 3), 1371 (40.4%) assuming deaths would be reduced proportionally to the number of categories implemented, and 1862 (54.9%) assuming any single category implemented among categories recommended would have sufficed to prevent the death.

Of 2607 deaths deemed preventable across all sites and age groups, for 1887 (72.3%) DECODE panelists also recommended specific public health actions beyond the 10 categories, most of which pertained to improvements in clinical management and quality of care. Timely caesarian delivery (recommended to prevent 79 of 633 stillbirths [12.5%]), management of hypertension (to prevent 79 stillbirths [12.5%]), improvements in medical records such as documenting hypertension or HIV infection in the mother (to prevent 75 stillbirths [11.8%]), and folic acid fortification (to prevent 55 stillbirths [8.7%]) were the most frequent clinical management recommendations (eTable 4 in Supplement 1). Most cited for neonatal deaths were improved hygiene and sanitation (to prevent 169 of 725 neonatal deaths [23.3%]), advanced respiratory support (to prevent 84 neonatal deaths [11.6%]), improvements in medical records (to prevent 73 neonatal deaths [10.1%]), and appropriate use of antibiotics (to prevent 71 neonatal deaths [9.8%]). Most cited for infants and children were nutritional support (to prevent 110 of 529 infant and child deaths [20.8%]), management of HIV (to prevent 74 infant and child deaths [14.0%]), hygiene and sanitation (to prevent 73 infant and child deaths [13.8%]), and clinical laboratory services (to prevent 52 infant and child deaths [9.8%]). Public health actions were originally intended to be used as a catchall and not tied to any single health system improvement category, so there may be some overlap between specific public health actions (eFigure 6 in Supplement 1).

Of the 1887 deaths for which public health actions were provided beyond the 10 categories, for 700 (37.1%) the panel reiterated that improved health-seeking behavior or sensitization to danger signs during pregnancy could have prevented the death. Furthermore, the panel suggested 122 deaths (6.5%) could have been prevented with education on proper diet, 70 (3.7%) by reduced reliance on herbal or traditional medicine, 49 (2.6%) by use of bed nets and improved environmental sanitation to prevent malaria, 23 (1.2%) by adequate breastfeeding or weaning, and 21 (1.1%) by understanding the dangers of over-the-counter medication (data not presented).

## Discussion

From an analysis of 3390 deaths in 7 countries with high mortality rates among children younger than 5 years, improved clinical management and quality of care as well as ANC and obstetric care could have prevented the most stillbirths and neonatal deaths. For infants and children, most deaths could have been prevented with improved clinical management, infection prevention and control, or health education. These categories may guide selection of interventions targeting common causes of deaths among newborns and children younger than 5 years to achieve SDG 2030 goals for reducing child mortality.

Prior studies suggest maternal undernutrition, obesity, diabetes, stress, depression, smoking, alcohol use, or drug consumption may increase risk of infections, preterm birth, and/or congenital malformations.^17^ Our study demonstrates that regular ANC visits may be the most critical measure to prevent the most stillbirths and neonatal deaths. At least 4 ANC visits may reduce leading causes of neonatal deaths.^18,19^ Ultrasonography access, timely caesarian delivery, trained staff for parturition, fetal heart rate monitoring, and improving maternal nutrition were among recommendations to improve ANC and obstetric care in our study, which is consistent with other studies.^20^ Beyond what can be prevented with existing modalities, CHAMPS data also provide insights into what new tools are needed. Considering the contribution of neonatal sepsis to mortality, it is likely that development and use of interventions including maternal immunization, rapid diagnostics, and novel therapeutics would substantially reduce neonatal mortality. A systematic review highlighted measures to reduce mortality among preterm and low-birth-weight neonates in low- and middle-income countries.^21^ WHO adopted 10 main recommendations to improve preterm birth outcomes, which include when to use antenatal corticosteroid therapy, tocolysis, magnesium sulfate for neuroprotection, antibiotics for premature rupture of membranes, mode of preterm birth, kangaroo mother care, plastic wraps, continuous positive airway pressure therapy, and surfactant and oxygen therapy.^22^ Adoption of these interventions, however, may result in prolonged hospitalization and subsequent risk for hospital-acquired infection. Low-cost antenatal, intrapartum, and postnatal interventions could prevent up to 28% of neonatal deaths, 22% of stillbirths, and 28% of maternal deaths each year.^23^ Studies have found that investments in prenatal care, such as those listed in the WHO guidelines, could triple the return on investment in terms of lives saved.^24^

Perinatal asphyxia or hypoxia was the most frequent cause of death for stillbirths among all sites and for neonatal deaths among 4 sites. Preterm infants are at high risk for respiratory distress because of underdeveloped lungs; interventions such as ventilators and antenatal corticosteroids can stabilize breathing and help the lungs mature more quickly.^25^ Resuscitating newborns with ambient air could prevent up to 30% of asphyxia deaths.^26^ Programmatic interventions, including training birth attendants with, eg, Helping Babies Breathe (implemented in CHAMPS countries), have demonstrated significant reductions in neonatal mortality.^27,28,29^ Novel surfactants containing synthetic phospholipid with peptide analogs of surfactant protein B and C are being evaluated to improve pulmonary outcomes.^30^

Improved clinical management and quality of care was recommended to prevent more than one-third of all deaths. Health care facilities can be interrupted by power or water shortages and are often underresourced in terms of medical supplies, diagnostic and therapeutic tools, and trained health care professionals.^31^ Improving medical recordkeeping was frequently cited to prevent deaths. Better documentation of clinical examinations, procedures, and test results would enable proper follow-up of patients with hypertension, HIV, and other conditions. Improving patient triage and referrals was also recommended to prevent many deaths, given that health care facilities are often challenged by overcrowded services, lack of privacy, long waiting times, delayed referrals, administrative and financial challenges, and lack of guidelines or standards of care and regulation.^32^ Many infants and children in this study died from multiple causes, presenting difficulties for diagnosis and treatment. WHO developed guidelines for the management of common childhood illnesses at the first-referral level in low-resource countries.^33^

Malnutrition was a leading cause of death according to DECODE panels among infants and children from all sites. Recommendations to prevent related deaths included improved nutritional support, health education, and clinical management. Myriad factors may contribute to malnutrition, including quality of diet, maternal health, poverty, infectious disease, natural disasters, and war and conflict.^34,35^ The UN General Assembly proclaimed 2016 to 2025 the United Nations Decade of Action on Nutrition, calling for universal access to nutrition interventions and healthy diets from sustainable food systems.^36^ Specific interventions, such as rehydration therapy and vitamin A or zinc supplementation, may reduce mortality from childhood diarrhea and pneumonia.^37^

IPC was most often recommended to prevent sepsis, which was among the 4 leading causes of all neonatal and infant and child deaths in all sites. Sepsis can occur from unsanitary conditions at birth, infections transmitted during pregnancy, or nosocomial infections. Sepsis morbidity and mortality may be reduced with appropriate antibiotics linked with sensitivity testing, vaccinations, IPC, and health care quality improvement.^38,39^ For some causal pathogens (eg, *Klebsiella pneumoniae*, *Acinetobacter baumannii*), which are highly drug resistant, novel therapeutics, vaccines, and diagnostic tools are needed.

Malaria was a leading cause of death among infants and children in Kenya, Sierra Leone, and Mozambique. Recommendations to prevent malaria deaths included improved clinical management, health-seeking behavior, and health education. Early diagnosis and treatment with artemisinin-based combination therapy reduces disease severity and prevents further transmission.^40,41^ Health education, including appropriate and consistent use of insecticide-treated bed nets, may reduce stillbirths or miscarriages and child mortality.^42,43^ Recently, RTS,S malaria vaccines have shown some reduction of severe malaria in children^44^ but have only been recently recommended for widespread use in malaria-endemic settings of sub-Saharan Africa.

### Limitations

This study has several limitations. First, it focused on broad health system improvement categories, which included a range of interventions. For example, improvements in HIV prevention and control include greater maternal access to testing and antiretroviral therapy, therapy for neonatal infections, and others. The categories were developed from recommendations from the first DECODE panels and are being refined to be more specific. Second, despite efforts to standardize procedures, myriad factors preclude our ability to make direct comparisons between sites, including differences in catchment area populations, consent rates, clinical care, and diagnostic capabilities, and others. Third, less than half of eligible deaths at CHAMPS sites undergo MITS, so the numbers of deaths from specific causes may not be representative of all deaths from those sites. Deaths in the CHAMPS catchment areas are required to be identified within 24 hours (72 hours if refrigerated) to qualify for MITS, favoring inclusion of deaths from health care facilities vs from communities. Fourth, the MITS procedure has low sensitivity for detection of deaths caused by trauma, congenital abnormalities, and genetic disorders. The DECODE process of determining preventability used open-text fields to list public health actions, which could be harmonized by providing a drop-down menu of specific public health and clinical actions. Notwithstanding these limitations, our study included information from the entire causal chain of mortality, determined by expert panels informed by objective clinical, pathological, and laboratory findings,^12^ to a degree not previously available to better characterize deaths.

## Conclusions

In this study, most childhood deaths in the CHAMPS network were deemed preventable when considering the immediate circumstances surrounding the child’s death. Investments in interventions that reduce child mortality should focus on improvements in antenatal care and higher quality of pediatric care. Coordinated efforts are needed by governments, nongovernmental organizations, the private sector, the community, and other stakeholders to articulate and implement rational, evidence-based public health care policies to reduce child mortality. Beyond clinical care, broader political, social, and financial reforms will further reduce child mortality. Targeted efforts to combat malnutrition, malaria, and infections are important areas for investment. Concomitant with disease-specific interventions, effective socioeconomic interventions, such as increasing access to family planning, improving education, reducing poverty, and strengthening health care systems, can also reduce child mortality.^45^

## Notes

Supplement 1. **eFigure 1. **Flowchart of Enrolled Deaths From CHAMPS Sites Between December 2016 and December 2021 With MITS and Consent Only for Verbal Autopsy and Clinical Abstraction (Non-MITS) and Were Included in the Analysis **eFigure 2. **Proportion of the 5 Most Frequent Causes of Death Anywhere in the Causal Pathway That Were Deemed Preventable by the Expert Determination of Cause of Death Panel for Neonatal and Infant and Child Deaths, December 2016 to December 2021 **eFigure 3. **Most Frequent Causes of Death Anywhere in the Causal Chain for Which the Optimal Health System Improvement Categories Were Recommended to Prevent Death for Each Age Group, December 2016 to December 2021 **eFigure 4. **Optimal Combinations of Health System Improvement Categories That Could Have Prevented the Most Deaths Across All Sites by Age Group, December 2016 to December 2021 **eFigure 5. **Proportion of the 5 Most Frequent Causes of Death That Could Have Been Prevented for the Optimal Combinations of Health System Improvement Categories That Could Have Prevented the Most Deaths Across All Sites by Age Group, December 2016 to December 2021 **eFigure 6. **Alluvial Plot of the Top 20 Most Recommended Interventions Associated With Improved Clinical Management or Improved Antenatal and Obstetric Care Across All Age Groups, December 2016 to December 2021 **eTable 1. **Characteristics of Deaths Enrolled in CHAMPS, December 2016 to December 2021 **eTable 2. **The 10 Most Frequent Causes of Death by Age Group and Site, December 2016 to December 2021 **eTable 3. **Proportion of Deaths That Were Deemed Preventable by Age Group and Site, December 2016 to December 2021 **eTable 4. **Specific Public Health Actions Recommended by the Expert Determination of Cause of Death Panel to Prevent Deaths by Age Group **eTable 5. **Number and Proportion of Deaths of Any Cause That Could Have Been Prevented for Each Single Health System Improvement Category Assuming (A1) All Recommendations Given for a Single Death Are Necessary to Prevent That Death, (A2) Deaths Would be Reduced Proportionally for Every Category Implemented, and (A3) Any Single Category Among All Categories Recommended for Each Death Is Sufficient to Prevent that Death, by Site and Age Group, December 2016 to December 2021 **eTable 6. **Number and Proportion of Deaths That Could Have Been Prevented From the Optimal Combinations of Health System Improvement Categories Assuming All Recommendations Given for a Single Death Are Necessary to Prevent That Death by Site and Age Group, December 2016 to December 2021 **eTable 7. **The Number and Proportion of Deaths for Each of the Top 5 Causes of Death for Each Age Group That Could Have Been Prevented for the Top Combination of 1 to 3 Health System Improvement Categories, Assuming All the Recommendations Given for a Single Death Are Necessary to Prevent That Death and the Death Would Be Prevented When Any Single Cause of Death Was Prevented, December 2016 to December 2021 **eTable 8. **The Number and Proportion of Deaths for Each of the Top 5 Causes of Death for Each Age Group That Could Have Been Prevented for the Top Combination of 1 to 3 Health System Improvement Categories, Assuming All the Recommendations Given for a Single Death Are Necessary to Prevent That Death, With Deaths Weighted by Giving 50% to the Underlying Cause With the Remaining Weight Distributed Evenly Among the Other Causes, December 2016 to December 2021 **eTable 9. **The Number and Proportion of Deaths for Each of the Top 5 Causes of Death for Each Age Group That Could Have Been Prevented for the Top Combination of 1 to 3 Health System Improvement Categories, Assuming All the Recommendations Given for a Single Death Are Necessary to Prevent That Death, With Deaths Weighted Using a Causal Chain Approach With Greater Weight Given to Underlying, Comorbid, and Immediate Causes of Death, Respectively, December 2016 to December 2021 **eMethods. **Statistical Analysis

Supplement 2.
Nonauthor Collaborators
