# Supplementary material for: Prioritizing Health Care Strategies to Reduce Childhood Mortality
Source: JAMA Netw Open. 2022 Oct 21;5(10):e2237689. doi: 10.1001/jamanetworkopen.2022.37689 (PMC9587481; doi:10.1001/jamanetworkopen.2022.37689)
Supplement: Supplement 1. — eFigure 1. Flowchart of Enrolled Deaths From CHAMPS Sites Between December 2016 and December 2021 With MITS and Consent Only for Verbal Autopsy and Clinical Abstraction (Non-MITS) and Were Included in the Analysis eFigure 2. Proportion of the 5 Most Frequent Causes of Death Anywhere in the Causal Pathway That Were Deemed Preventable by the Expert Determination of Cause of Death Panel for Neonatal and Infant and Child Deaths, December 2016 to December 2021 eFigure 3. Most Frequent Causes of Death Anywhere in the Causal Chain for Which the Optimal Health System Improvement Categories Were Recommended to Prevent Death for Each Age Group, December 2016 to December 2021 eFigure 4. Optimal Combinations of Health System Improvement Categories That Could Have Prevented the Most Deaths Across All Sites by Age Group, December 2016 to December 2021 eFigure 5. Proportion of the 5 Most Frequent Causes of Death That Could Have Been Prevented for the Optimal Combinations of Health System Improvement Categories That Could Have Prevented the Most Deaths Across All Sites by Age Group, December 2016 to December 2021 eFigure 6. Alluvial Plot of the Top 20 Most Recommended Interventions Associated With Improved Clinical Management or Improved Antenatal and Obstetric Care Across All Age Groups, December 2016 to December 2021 eTable 1. Characteristics of Deaths Enrolled in CHAMPS, December 2016 to December 2021 eTable 2. The 10 Most Frequent Causes of Death by Age Group and Site, December 2016 to December 2021 eTable 3. Proportion of Deaths That Were Deemed Preventable by Age Group and Site, December 2016 to December 2021 eTable 4. Specific Public Health Actions Recommended by the Expert Determination of Cause of Death Panel to Prevent Deaths by Age Group eTable 5. Number and Proportion of Deaths of Any Cause That Could Have Been Prevented for Each Single Health System Improvement Category Assuming (A1) All Recommendations Given for a Single Death Are Necessary to Prevent That Death, (A2) [file jamanetwopen-e2237689-s001.pdf]

Supplemental Online Content

Madewell ZJ, Whitney CG, Velaphi S, et al; Child Health and Mortality Prevention Surveillance Network. Prioritizing health care strategies to reduce childhood mortality. *JAMA Netw Open.* 2022;5(10):e2237689. doi:10.1001/jamanetworkopen.2022.37689

eFigure 1. Flowchart of Enrolled Deaths From CHAMPS Sites Between December 2016 and December 2021 With MITS and Consent Only for Verbal Autopsy and Clinical Abstraction (Non-MITS) and Were Included in the Analysis

eFigure 2. Proportion of the 5 Most Frequent Causes of Death Anywhere in the Causal Pathway That Were Deemed Preventable by the Expert Determination of Cause of Death Panel for Neonatal and Infant and Child Deaths, December 2016 to December 2021

eFigure 3. Most Frequent Causes of Death Anywhere in the Causal Chain for Which the Optimal Health System Improvement Categories Were Recommended to Prevent Death for Each Age Group, December 2016 to December 2021

eFigure 4. Optimal Combinations of Health System Improvement Categories That Could Have Prevented the Most Deaths Across All Sites by Age Group, December 2016 to December 2021

eFigure 5. Proportion of the 5 Most Frequent Causes of Death That Could Have Been Prevented for the Optimal Combinations of Health System Improvement Categories That Could Have Prevented the Most Deaths Across All Sites by Age Group, December 2016 to December 2021

eFigure 6. Alluvial Plot of the Top 20 Most Recommended Interventions Associated With Improved Clinical Management or Improved Antenatal and Obstetric Care Across All Age Groups, December 2016 to December 2021

eTable 1. Characteristics of Deaths Enrolled in CHAMPS, December 2016 to December 2021

eTable 2. The 10 Most Frequent Causes of Death by Age Group and Site, December 2016 to December 2021

eTable 3. Proportion of Deaths That Were Deemed Preventable by Age Group and Site, December 2016 to December 2021

eTable 4. Specific Public Health Actions Recommended by the Expert Determination of Cause of Death Panel to Prevent Deaths by Age Group

eTable 5. Number and Proportion of Deaths of Any Cause That Could Have Been Prevented for Each Single Health System Improvement Category Assuming (A1) All Recommendations Given for a Single Death Are Necessary to Prevent That Death, (A2) Deaths Would be Reduced Proportionally for Every Category Implemented, and (A3) Any Single Category Among All Categories Recommended for Each Death Is Sufficient to Prevent that Death, by Site and Age Group, December 2016 to December 2021

eTable 6. Number and Proportion of Deaths That Could Have Been Prevented From the Optimal Combinations of Health System Improvement Categories Assuming All Recommendations Given for a Single Death Are Necessary to Prevent That Death by Site and Age Group, December 2016 to December 2021

eTable 7. The Number and Proportion of Deaths for Each of the Top 5 Causes of Death for Each Age Group That Could Have Been Prevented for the Top Combination of 1 to 3 Health System Improvement Categories, Assuming All the Recommendations Given for a Single Death Are Necessary to Prevent That Death and the Death Would Be Prevented When Any Single Cause of Death Was Prevented, December 2016 to December 2021

eTable 8. The Number and Proportion of Deaths for Each of the Top 5 Causes of Death for Each Age Group That Could Have Been Prevented for the Top Combination of 1 to 3 Health System Improvement Categories,

© 2022 Madewell ZJ et al. *JAMA Network Open.*

Assuming All the Recommendations Given for a Single Death Are Necessary to Prevent That Death, With Deaths Weighted by Giving 50% to the Underlying Cause With the Remaining Weight Distributed Evenly Among the Other Causes, December 2016 to December 2021

eTable 9. The Number and Proportion of Deaths for Each of the Top 5 Causes of Death for Each Age Group That Could Have Been Prevented for the Top Combination of 1 to 3 Health System Improvement Categories, Assuming All the Recommendations Given for a Single Death Are Necessary to Prevent That Death, With Deaths Weighted Using a Causal Chain Approach With Greater Weight Given to Underlying, Comorbid, and Immediate Causes of Death, Respectively, December 2016 to December 2021

eMethods. Statistical Analysis

This supplemental material has been provided by the authors to give readers additional information about their work.

eFigure 1. Flowchart of Enrolled Deaths From CHAMPS Sites Between December 2016 and December 2021 With MITS and Consent Only for Verbal Autopsy and Clinical Abstraction (Non-MITS) and Were Included in the Analysis

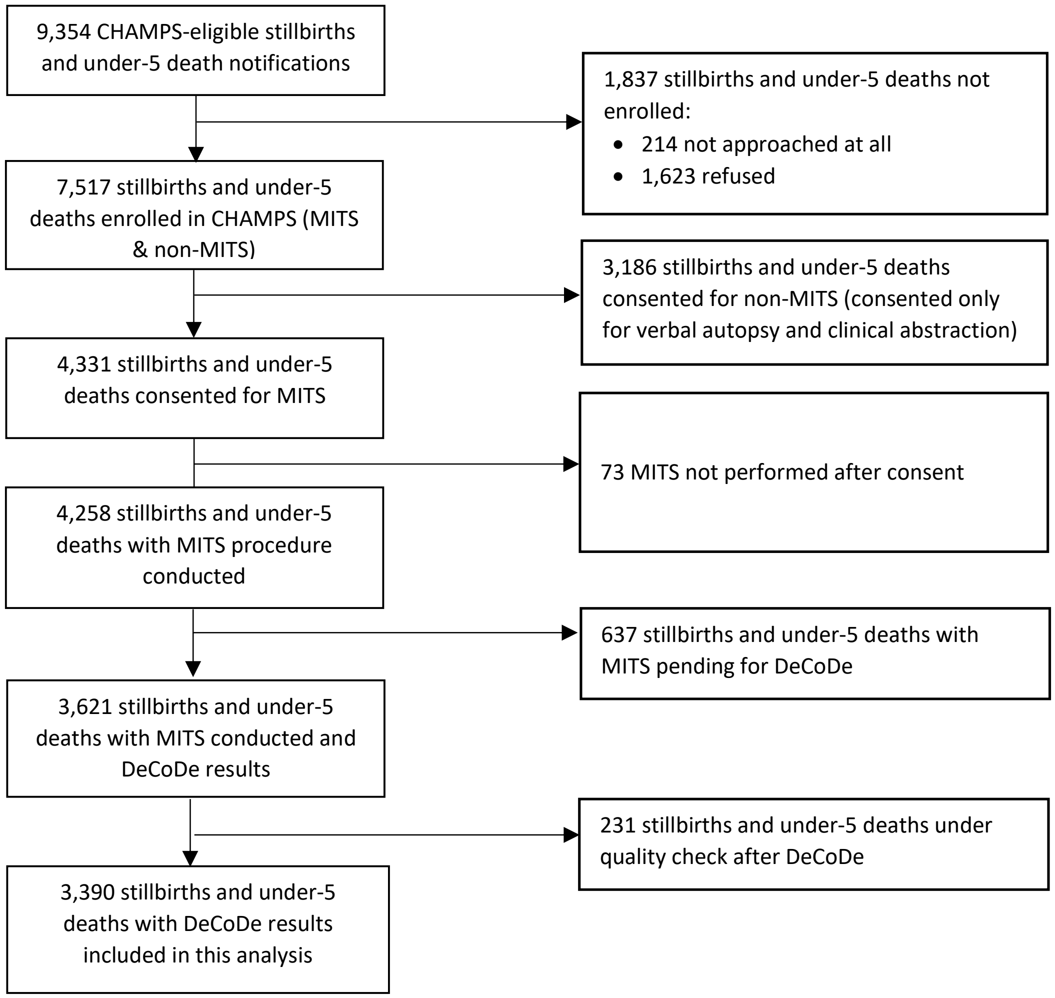

© 2022 Madewell ZJ et al. *JAMA Network Open.*

eFigure 2. Proportion of the 5 Most Frequent Causes of Death Anywhere in the Causal Pathway That Were Deemed Preventable by the Expert Determination of Cause of Death Panel for Neonatal and Infant and Child Deaths, December 2016 to December 2021

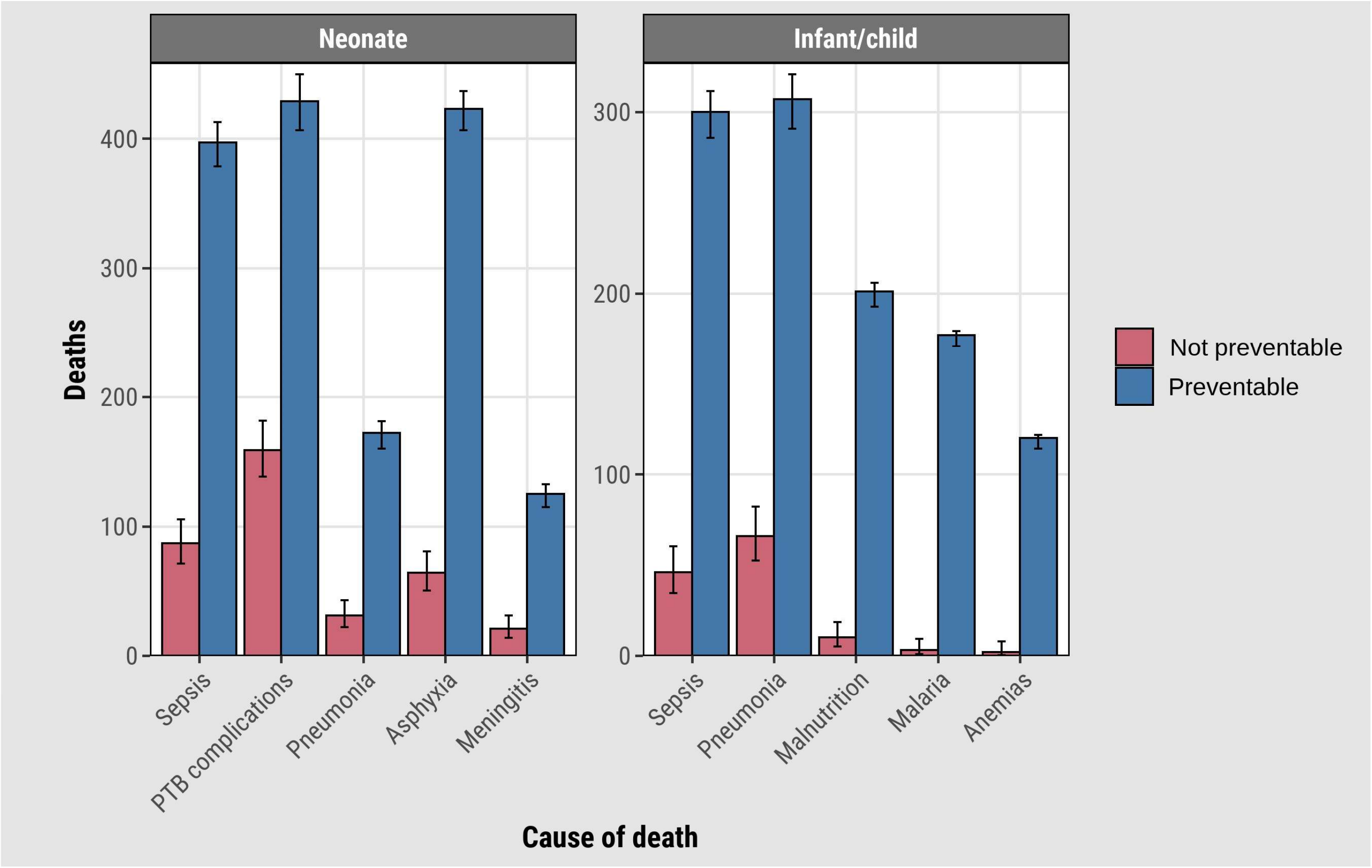

© 2022 Madewell ZJ et al. *JAMA Network Open.*

eFigure 3. Most Frequent Causes of Death Anywhere in the Causal Chain for Which the Optimal Health System Improvement Categories Were Recommended to Prevent Death for Each Age Group, December 2016 to December 2021

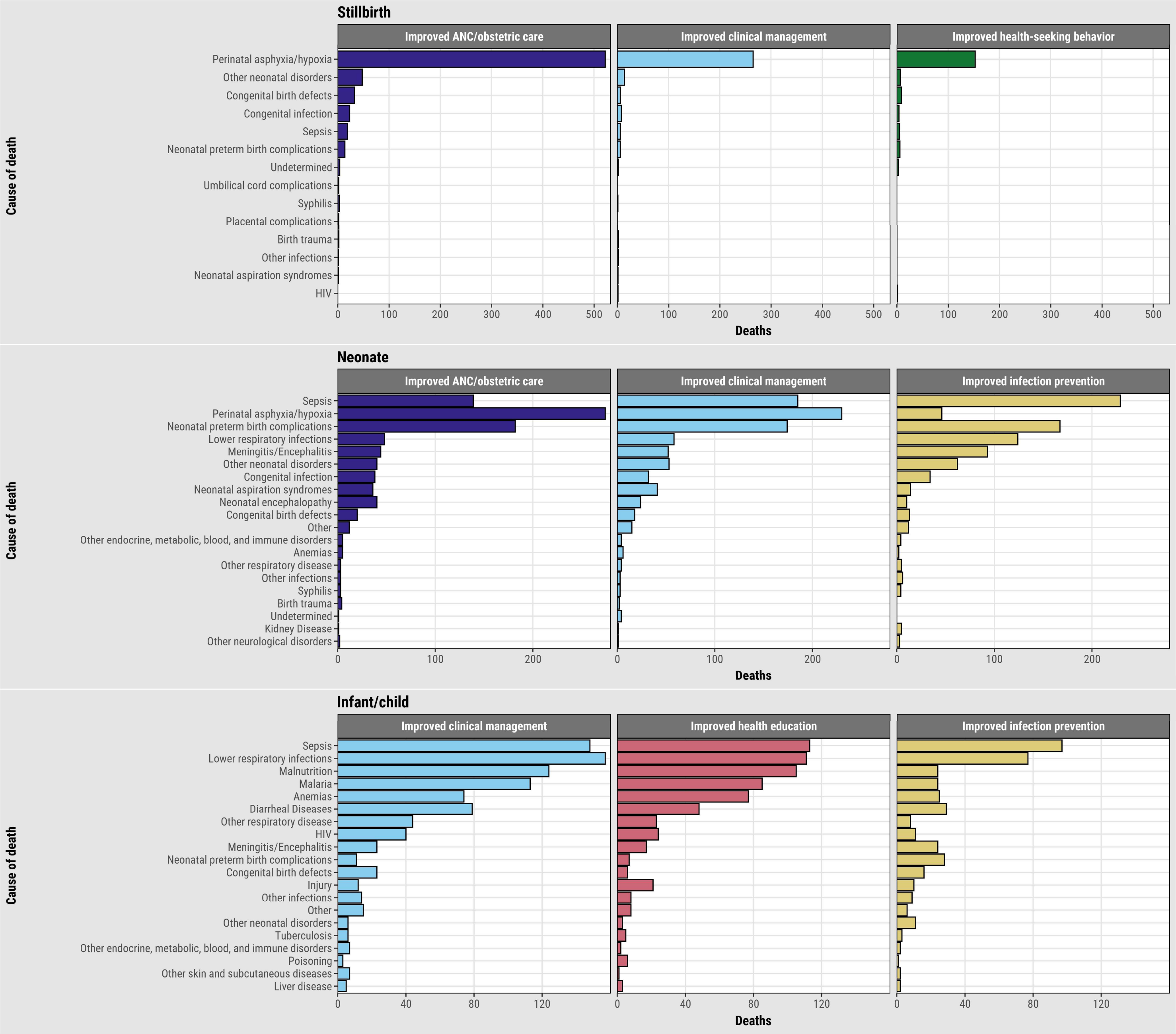

© 2022 Madewell ZJ et al. *JAMA Network Open.*

eFigure 4. Optimal Combinations of Health System Improvement Categories That Could Have Prevented the Most Deaths Across All Sites by Age Group, December 2016 to December 2021

This figure assumes all recommendations given for a single death are necessary to prevent that death. The stacked bars represent the proportion of additional deaths that could have been prevented with each additional health system improvement category implemented.

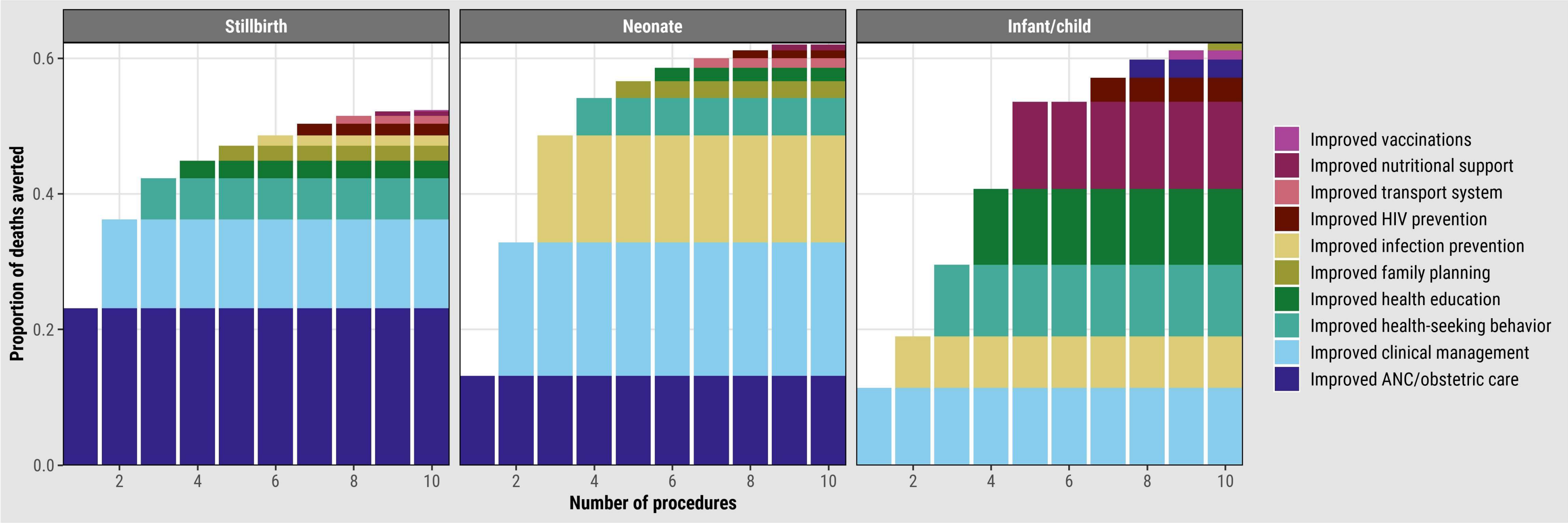

Examining which combinations of 1-10 health system improvement categories could have prevented the most deaths demonstrates that most preventable stillbirths or neonatal deaths could have been prevented with three categories improved and most infant/child deaths could have been prevented with five categories improved under the assumption that all categories given for a single death are necessary to prevent that death.

© 2022 Madewell ZJ et al. *JAMA Network Open.*

eFigure 5. Proportion of the 5 Most Frequent Causes of Death That Could Have Been Prevented for the Optimal Combinations of Health System Improvement Categories That Could Have Prevented the Most Deaths Across All Sites by Age Group, December 2016 to December 2021

This plot assumes all recommendations given for a single death are necessary to prevent that death.

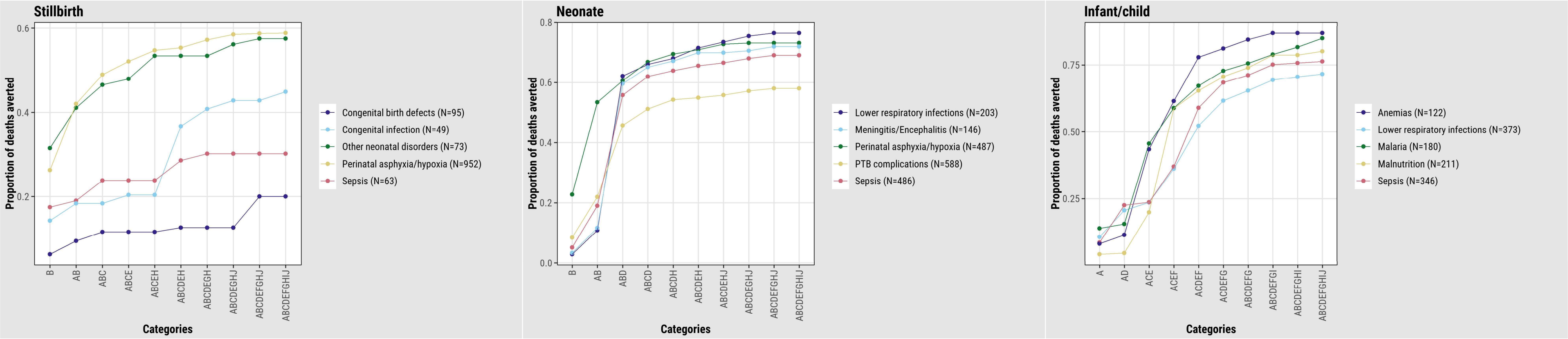

A: Improved clinical management and quality of care
B: Improved antenatal and obstetric care and management
C: Improved health-seeking behavior
D: Improved infection prevention and control
E: Improved health education [immunizations, preventing malnutrition, diarrhea, malaria, burns, poisoning, etc.]
F: Improved nutritional support
G: Improved HIV prevention and control
H: Improved family planning
I: Improved use of existing vaccinations
J: Improved transport system

© 2022 Madewell ZJ et al. *JAMA Network Open.*

eFigure 6. Alluvial Plot of the Top 20 Most Recommended Interventions Associated With Improved Clinical Management or Improved Antenatal and Obstetric Care Across All Age Groups, December 2016 to December 2021

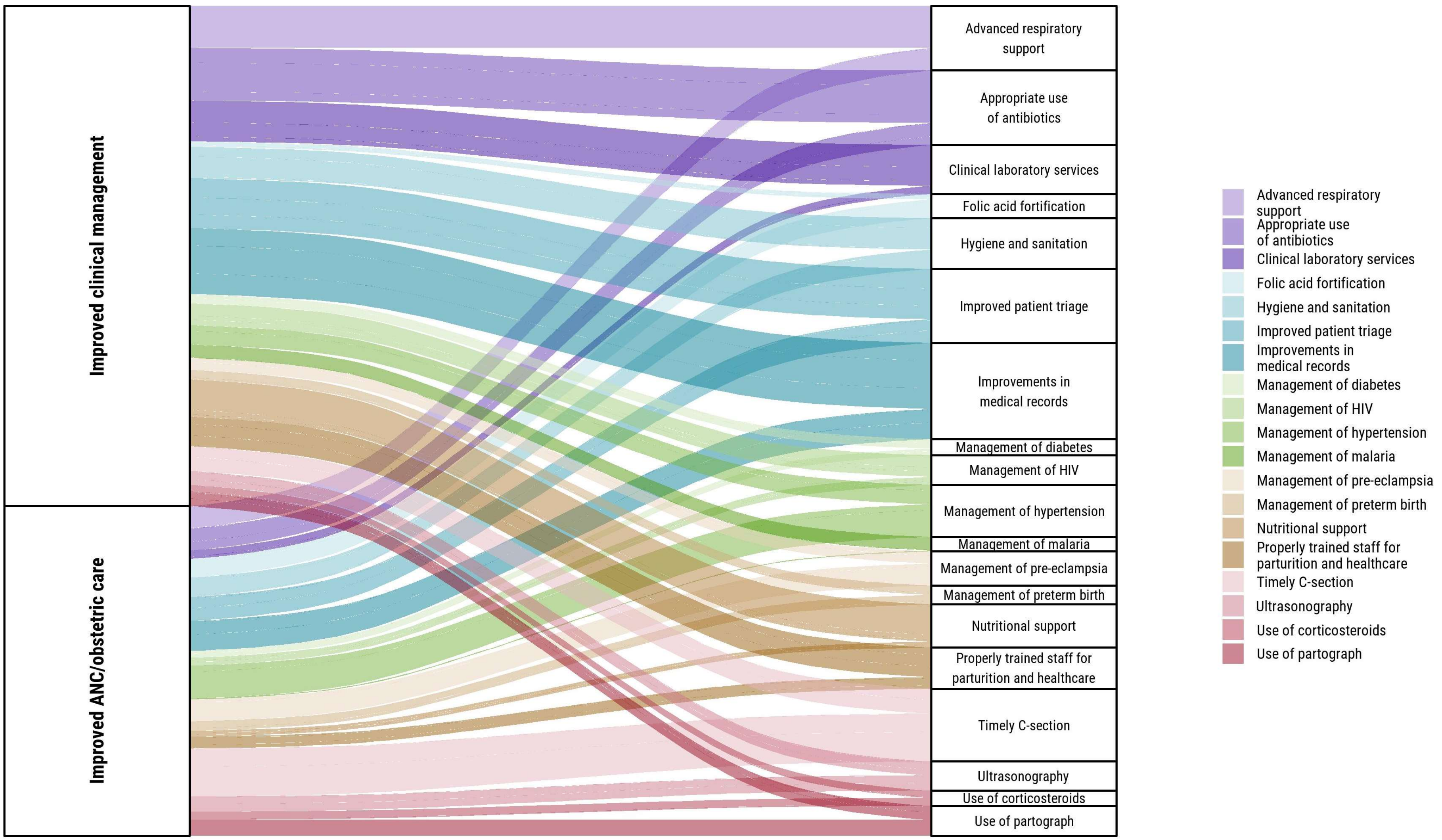

.

© 2022 Madewell ZJ et al. *JAMA Network Open.*

eTable 1. Characteristics of Deaths Enrolled in CHAMPS, December 2016 to December 2021			
	**Stillbirth**	**Neonate**	**Infant/child**
	**N=1,190**	**N=1,340**	**N=860**
Characteristic	**N (%)**	**N (%)**	**N (%)**
CHAMPS sites			
Bangladesh	183 (15.4)	177 (13.2)	4 (0.5)
Ethiopia	207 (17.4)	101 (7.5)	26 (3.0)
Kenya	167 (14.0)	173 (12.9)	238 (27.7)
Mali	82 (6.9)	74 (5.5)	52 (6.0)
Mozambique	256 (21.5)	255 (19.0)	143 (16.6)
Sierra Leone	120 (10.1)	124 (9.3)	176 (20.5)
South Africa	175 (14.7)	436 (32.5)	221 (25.7)
Sex			
Female	538 (45.2)	576 (43.0)	391 (45.5)
Male	650 (54.6)	761 (56.8)	469 (54.5)
Indeterminate	2 (0.2)	3 (0.2)	0 (0)
Median (IQR) weight at MITS, in grams (N=3,369)	2,200 (1,500, 2,900)	1,808 (1,100, 2,800)	6,955 (4,316, 10,000)
Weight for height z-score, n (%)			
Normal weight for height (≥ -2SD)	--	297 (22.2)	354 (41.2)
Moderate wasting (<-2 SD, -3 SD)	--	92 (6.9)	120 (14.0)
Severe wasting (<-3 SD)	--	234 (17.5)	325 (37.8)
Not recorded	--	717 (53.5)	61 (7.1)
Weight for age z-score, n (%)			
Normal weight for age (≥-2SD)	--	462 (34.5)	332 (38.6)
Moderate underweight (<-2 SD, -3 SD)	--	110 (8.2)	135 (15.7)
Severe underweight (<-3 SD)	--	757 (56.5)	382 (44.4)
Not recorded	--	11 (0.8)	11 (1.3)
Height for age z-score, n (%)			
Normal height for age (≥-2SD)	--	516 (38.5)	494 (57.4)
Moderate stunting (<-2 SD, -3 SD)	--	103 (7.7)	123 (14.3)
Severe stunting (<- 3 SD)	--	714 (53.3)	241 (28.0)
Not recorded	--	7 (0.5)	2 (0.2)
Median (IQR) of hours between death and MITS done (N=3,375)	10 (3, 19)	12 (4, 21)	14 (6, 21)
Location of death, n (%) (N=3,392)			
Community	41 (3.4)	68 (5.1)	235 (27.3)
Health facility	1,148 (96.6)	1,272 (94.9)	625 (72.7)

© 2022 Madewell ZJ et al. *JAMA Network Open.*

eTable 2. The 10 Most Frequent Causes of Death by Age Group and Site, December 2016 to December 2021							
Cause of death	**Bangladesh**	**Ethiopia**	**Kenya**	**Mali**	**Mozambique**	**Sierra Leone**	**South Africa**
	**N (%)**	**N (%)**	**N (%)**	**N (%)**	**N (%)**	**N (%)**	**N (%)**
Stillbirth	N=183	N=207	N=167	N=82	N=256	N=120	N=175
Perinatal asphyxia/hypoxia	169 (92.3)	143 (69.1)	156 (93.4)	70 (85.4)	207 (80.9)	107 (89.2)	100 (57.1)
Congenital birth defects	10 (5.5)	49 (23.7)	0 (0)	8 (9.8)	15 (5.9)	6 (5.0)	7 (4.0)
Other neonatal disorders	38 (20.8)	15 (7.2)	2 (1.2)	13 (15.9)	1 (0.4)	3 (2.5)	1 (0.6)
Sepsis	2 (1.1)	6 (2.9)	2 (1.2)	3 (3.7)	5 (2.0)	1 (0.8)	44 (25.1)
Congenital infection	7 (3.8)	0 (0)	3 (1.8)	0 (0)	23 (9.0)	2 (1.7)	14 (8.0)
Neonatal preterm birth complications	11 (6.0)	1 (0.5)	0 (0)	0 (0)	2 (0.8)	10 (8.3)	0 (0)
Syphilis	0 (0)	0 (0)	0 (0)	0 (0)	3 (1.2)	0 (0)	3 (1.7)
Other infections	3 (1.6)	0 (0)	0 (0)	0 (0)	0 (0)	0 (0)	1 (0.6)
Neonatal aspiration syndromes	2 (1.1)	0 (0)	1 (0.6)	0 (0)	0 (0)	0 (0)	0 (0)
Placental complications	0 (0)	0 (0)	0 (0)	2 (2.4)	0 (0)	0 (0)	1 (0.6)
Neonate	N=177	N=10	N=173	N=74	N=255	N=124	N=436
Neonatal preterm birth complications	105 (59.3)	42 (41.6)	45 (26.0)	25 (33.8)	63 (24.7)	33 (26.6)	275 (63.1)
Perinatal asphyxia/hypoxia	109 (61.6)	38 (37.6)	61 (35.3)	16 (21.6)	106 (41.6)	76 (61.3)	81 (18.6)
Sepsis	46 (26.0)	63 (62.4)	37 (21.4)	27 (36.5)	53 (20.8)	40 (32.3)	218 (50.0)
Lower respiratory infections	2 (1.1)	29 (28.7)	10 (5.8)	15 (20.3)	16 (6.3)	10 (8.1)	121 (27.8)
Other neonatal disorders	26 (14.7)	19 (18.8)	6 (3.5)	7 (9.5)	5 (2.0)	2 (1.6)	99 (22.7)
Meningitis/Encephalitis	4 (2.3)	33 (32.7)	2 (1.2)	4 (5.4)	4 (1.6)	12 (9.7)	87 (20.0)
Congenital birth defects	12 (6.8)	10 (9.9)	6 (3.5)	20 (27.0)	18 (7.1)	7 (5.6)	56 (12.8)
Congenital infection	6 (3.4)	10 (9.9)	3 (1.7)	2 (2.7)	22 (8.6)	13 (10.5)	47 (10.8)
Neonatal encephalopathy	18 (10.2)	2 (2.0)	2 (1.2)	7 (9.5)	10 (3.9)	1 (0.8)	37 (8.5)
Neonatal aspiration syndromes	4 (2.3)	22 (21.8)	14 (8.1)	3 (4.1)	7 (2.7)	8 (6.5)	13 (3.0)
Infant/child	N=4	N=26	N=238	N=52	N=143	N=176	N=221
Lower respiratory infections	1 (25.0)	20 (76.9)	69 (29.0)	26 (50.0)	70 (49.0)	60 (34.1)	127 (57.5)
Sepsis	3 (75.0)	21 (80.8)	68 (28.6)	30 (57.7)	45 (31.5)	70 (39.8)	109 (49.3)
Malnutrition	1 (25.0)	18 (69.2)	76 (31.9)	14 (26.9)	12 (8.4)	77 (43.8)	13 (5.9)
Malaria	0 (0)	1 (3.8)	78 (32.8)	3 (5.8)	24 (16.8)	74 (42.0)	0 (0)
Anemias	1 (25.0)	2 (7.7)	4 (1.7)	1 (1.9)	9 (6.3)	105 (59.7)	0 (0)
Diarrheal Diseases	1 (25.0)	8 (30.8)	29 (12.2)	9 (17.3)	34 (23.8)	21 (11.9)	19 (8.6)
HIV	0 (0)	0 (0)	30 (12.6)	4 (7.7)	26 (18.2)	8 (4.5)	23 (10.4)
Congenital birth defects	1 (25.0)	0 (0)	16 (6.7)	6 (11.5)	11 (7.7)	0 (0)	45 (20.4)
Other respiratory disease	0 (0)	2 (7.7)	25 (10.5)	3 (5.8)	13 (9.1)	14 (8.0)	20 (9.0)
Meningitis/Encephalitis	0 (0)	11 (42.3)	2 (0.8)	6 (11.5)	2 (1.4)	9 (5.1)	38 (17.2)

© 2022 Madewell ZJ et al. *JAMA Network Open.*

eTable 3. Proportion of Deaths That Were Deemed Preventable by Age Group and Site, December 2016 to December 2021

	**Stillbirth (N=1,190)**	**Neonate (N=1,340)**	**Infant/child (N=860)**
Site	**N (%)**	**N (%)**	**N (%)**
Bangladesh	172 (94.0)	169 (95.5)	3 (75.0)
Ethiopia	196 (94.7)	98 (97.0)	26 (100.0)
Kenya	161 (96.4)	164 (94.8)	229 (96.2)
Mali	58 (70.7)	54 (73.0)	40 (76.9)
Mozambique	151 (59.0)	168 (65.9)	112 (78.3)
Sierra Leone	108 (90.0)	116 (93.5)	169 (96.0)
South Africa	37 (21.1)	241 (55.3)	135 (61.1)

© 2022 Madewell ZJ et al. *JAMA Network Open.*

eTable 4. Specific Public Health Actions Recommended by the Expert Determination of Cause of Death Panel to Prevent Deaths by Age Group			
	**Stillbirth (N=633)**	**Neonate (N=725)**	**Infant/child (N=529)**
Public health action	**N (%)**	**N (%)**	**N (%)**
Hygiene and sanitation	5 (0.8)	169 (23.3)	73 (13.8)
Improvements in medical records	75 (11.8)	73 (10.1)	45 (8.5)
Improved patient triage	51 (8.1)	52 (7.2)	49 (9.3)
Appropriate use of antibiotics	34 (5.4)	71 (9.8)	35 (6.6)
Nutritional support	13 (2.1)	12 (1.7)	110 (20.8)
Timely C-section	79 (12.5)	54 (7.4)	1 (0.2)
Advanced respiratory support	1 (0.2)	84 (11.6)	21 (4.0)
Management of HIV	17 (2.7)	14 (1.9)	74 (14.0)
Clinical laboratory services	14 (2.2)	34 (4.7)	52 (9.8)
Management of hypertension	79 (12.5)	19 (2.6)	0 (0.0)
Properly trained staff for parturition and healthcare	28 (4.4)	35 (4.8)	19 (3.6)
Folic acid fortification	55 (8.7)	12 (1.7)	3 (0.6)
Management of pre-eclampsia	45 (7.1)	12 (1.7)	0 (0.0)
Ultrasonography	25 (3.9)	30 (4.1)	2 (0.4)
Use of partograph	30 (4.7)	20 (2.8)	3 (0.6)
Management of malaria	2 (0.3)	0 (0.0)	31 (5.9)
Childhood vaccinations	0 (0.0)	2 (0.3)	30 (5.7)
Management of diabetes	17 (2.7)	11 (1.5)	3 (0.6)
Appropriate use of corticosteroids	4 (0.6)	21 (2.9)	1 (0.2)
Management of preterm birth	6 (0.9)	20 (2.8)	0 (0.0)
Availability of 24/7 healthcare services	8 (1.3)	7 (1.0)	10 (1.9)
Management of diarrheal diseases and rehydration	0 (0.0)	4 (0.6)	20 (3.8)
Radiography	5 (0.8)	8 (1.1)	9 (1.7)
Proper care of twin pregnancy	9 (1.4)	10 (1.4)	1 (0.2)
Fetal heart rate monitoring	12 (1.9)	5 (0.7)	2 (0.4)
Premature discharge	2 (0.3)	12 (1.7)	5 (0.9)
Proper management of maternal anemia during pregnancy	9 (1.4)	7 (1.0)	3 (0.6)
Sickle cell screening	4 (0.6)	3 (0.4)	12 (2.3)
Blood transfusion therapy	2 (0.3)	3 (0.4)	11 (2.1)
Management of burns	0 (0.0)	0 (0.0)	15 (2.8)
Prevention of hypothermia	0 (0.0)	12 (1.7)	2 (0.4)
Management of pneumonia	0 (0.0)	2 (0.3)	11 (2.1)
Group B streptococcus screening	5 (0.8)	6 (0.8)	0 (0.0)
Syphillis screening and treatment	6 (0.9)	4 (0.6)	1 (0.2)
Management of maternal trauma	6 (0.9)	3 (0.4)	1 (0.2)
Adherence to IMCI guidelines	0 (0.0)	1 (0.1)	8 (1.5)
Availability of medical equipment and supplies	1 (0.2)	7 (1.0)	1 (0.2)
Corrective surgical intervention	3 (0.5)	2 (0.3)	4 (0.8)

© 2022 Madewell ZJ et al. *JAMA Network Open.*

Management of anemia	6 (0.9)	0 (0.0)	3 (0.6)
Proper Apgar scoring	1 (0.2)	8 (1.1)	0 (0.0)
TORCH[b] screening	2 (0.3)	2 (0.3)	3 (0.6)
Proper administration of Oxytocin	3 (0.5)	3 (0.4)	0 (0.0)
Appropriate use of misoprostol	4 (0.6)	1 (0.1)	0 (0.0)
Management of shock	0 (0.0)	1 (0.1)	4 (0.8)
Antifungals	0 (0.0)	0 (0.0)	2 (0.4)
Availability of dermatologist	0 (0.0)	0 (0.0)	2 (0.4)
Management of congenital heart disease	0 (0.0)	1 (0.1)	1 (0.2)
Management of heart disease	0 (0.0)	2 (0.3)	0 (0.0)
Management of pertussis	0 (0.0)	0 (0.0)	2 (0.4)
More ICU beds	0 (0.0)	0 (0.0)	2 (0.4)
Pelvic measurement	2 (0.3)	0 (0.0)	0 (0.0)
Screening for cancer	0 (0.0)	0 (0.0)	2 (0.4)
Screening for tuberculosis	0 (0.0)	0 (0.0)	2 (0.4)
Availability of amniocentesis	0 (0.0)	1 (0.1)	0 (0.0)
Availability of chemotherapy drugs	0 (0.0)	0 (0.0)	1 (0.2)
Dental services	0 (0.0)	0 (0.0)	1 (0.2)
Management of Down syndrome	0 (0.0)	0 (0.0)	1 (0.2)
Rho(D) immune globulin	0 (0.0)	1 (0.1)	0 (0.0)
Screening for autoimmune diseases	1 (0.2)	0 (0.0)	0 (0.0)
Screening for jaundice	0 (0.0)	1 (0.1)	0 (0.0)
Treatment with Anti-D immunoglobulin	1 (0.2)	0 (0.0)	0 (0.0)

[a] Public health actions were originally intended to be used as a catch-all and not tied to any single health system improvement category, so there may be some overlap between specific public health actions (e.g., see eFigure 10 for example).

[b] TORCH: group of blood tests for toxoplasmosis, rubella cytomegalovirus, herpes simplex, and HIV.

© 2022 Madewell ZJ et al. *JAMA Network Open.*

eTable 5. Number and Proportion of Deaths of Any Cause That Could Have Been Prevented for Each Single Health System Improvement Category Assuming (A1) All Recommendations Given for a Single Death Are Necessary to Prevent That Death, (A2) Deaths Would be Reduced Proportionally for Every Category Implemented, and (A3) Any Single Category Among All Categories Recommended for Each Death Is Sufficient to Prevent that Death, by Site and Age Group, December 2016 to December 2021

Site	**Assumption**	**Improved ANC/obstetric care**	**Improved clinical management**	**Improved family planning**	**Improved health-seeking behavior**	**Improved health education**	**Improved HIV prevention**	**Improved infection prevention**	**Improved nutritional support**	**Improved transport system**	**Improved vaccinations**
		**N (%)**	**N (%)**	**N (%)**	**N (%)**	**N (%)**	**N (%)**	**N (%)**	**N (%)**	**N (%)**	**N (%)**
Stillbirth											
Bangladesh	A1	68 (37.2)	9 (4.9)	1 (0.5)	3 (1.6)	0 (0)	0 (0)	1 (0.5)	0 (0)	0 (0)	0 (0)
Bangladesh	A2	96 (52.2)	18 (10.1)	3 (1.5)	9 (4.8)	0 (0.0)	0 (0.0)	2 (1.3)	1 (0.5)	0 (0.0)	0 (0.3)
Bangladesh	A3	127 (69.4)	30 (16.4)	5 (2.7)	16 (8.7)	0 (0.0)	0 (0.0)	4 (2.2)	2 (1.1)	0 (0.0)	1 (0.5)
Ethiopia	A1	9 (4.3)	0 (0)	0 (0)	0 (0)	0 (0)	0 (0)	0 (0)	1 (0.5)	0 (0)	0 (0)
Ethiopia	A2	50 (24.3)	16 (7.6)	10 (4.7)	26 (12.6)	10 (4.9)	0 (0.2)	1 (0.3)	12 (5.6)	2 (1.0)	0 (0.0)
Ethiopia	A3	130 (62.8)	48 (23.2)	35 (16.9)	78 (37.7)	36 (17.4)	1 (0.5)	2 (1.0)	38 (18.4)	8 (3.9)	0 (0.0)
Kenya	A1	18 (10.8)	30 (18.0)	1 (0.6)	7 (4.2)	4 (2.4)	0 (0)	0 (0)	0 (0)	1 (0.6)	0 (0)
Kenya	A2	41 (24.5)	55 (33.2)	4 (2.6)	22 (13.0)	10 (6.2)	5 (3.2)	0 (0.1)	0 (0.0)	2 (0.9)	0 (0.0)
Kenya	A3	75 (44.9)	93 (55.7)	10 (6.0)	46 (27.5)	22 (13.2)	16 (9.6)	1 (0.6)	0 (0.0)	3 (1.8)	0 (0.0)
Mali	A1	36 (43.9)	6 (7.3)	0 (0)	0 (0)	1 (1.2)	0 (0)	0 (0)	0 (0)	1 (1.2)	0 (0)
Mali	A2	38 (46.3)	8 (9.8)	0 (0.0)	0 (0.0)	2 (1.8)	0 (0.0)	0 (0.0)	0 (0.0)	2 (1.8)	0 (0.0)
Mali	A3	40 (48.8)	10 (12.2)	0 (0.0)	0 (0.0)	2 (2.4)	0 (0.0)	0 (0.0)	0 (0.0)	2 (2.4)	0 (0.0)
Mozambique	A1	110 (43.0)	4 (1.6)	0 (0)	0 (0)	0 (0)	0 (0)	2 (0.8)	0 (0)	0 (0)	0 (0)
Mozambique	A2	122 (47.7)	14 (5.3)	0 (0.1)	2 (0.7)	1 (0.4)	0 (0.1)	7 (2.6)	0 (0.0)	2 (0.8)	0 (0.1)
Mozambique	A3	142 (55.5)	30 (11.7)	1 (0.4)	7 (2.7)	5 (2.0)	1 (0.4)	16 (6.2)	0 (0.0)	7 (2.7)	2 (0.8)
Sierra Leone	A1	11 (9.2)	16 (13.3)	0 (0)	10 (8.3)	0 (0)	1 (0.8)	0 (0)	0 (0)	0 (0)	0 (0)
Sierra Leone	A2	28 (23.3)	38 (31.4)	0 (0.1)	15 (12.8)	2 (2.1)	2 (1.4)	1 (0.6)	0 (0.4)	0 (0.0)	0 (0.0)
Sierra Leone	A3	47 (39.2)	61 (50.8)	1 (0.8)	21 (17.5)	7 (5.8)	3 (2.5)	2 (1.7)	1 (0.8)	0 (0.0)	0 (0.0)
South Africa	A1	23 (13.1)	5 (2.9)	0 (0)	0 (0)	0 (0)	2 (1.1)	1 (0.6)	0 (0)	0 (0)	0 (0)
South Africa	A2	25 (14.3)	6 (3.7)	0 (0.3)	0 (0.3)	0 (0.0)	2 (1.1)	1 (0.6)	0 (0.0)	0 (0.0)	0 (0.0)
South Africa	A3	27 (15.4)	8 (4.6)	1 (0.6)	1 (0.6)	0 (0.0)	2 (1.1)	1 (0.6)	0 (0.0)	0 (0.0)	0 (0.0)
Neonate											
Bangladesh	A1	34 (19.2)	32 (18.1)	1 (0.6)	1 (0.6)	0 (0)	0 (0)	2 (1.1)	0 (0)	0 (0)	0 (0)
Bangladesh	A2	67 (37.9)	60 (33.9)	2 (1.3)	10 (5.7)	0 (0.0)	0 (0.0)	8 (4.6)	1 (0.3)	0 (0.0)	0 (0.0)
Bangladesh	A3	107 (60.5)	96 (54.2)	4 (2.3)	23 (13.0)	0 (0.0)	0 (0.0)	18 (10.2)	2 (1.1)	0 (0.0)	0 (0.0)
Ethiopia	A1	1 (1.0)	1 (1.0)	0 (0)	0 (0)	0 (0)	0 (0)	0 (0)	0 (0)	0 (0)	0 (0)
Ethiopia	A2	24 (24.2)	28 (27.8)	3 (3.0)	11 (10.8)	2 (2.2)	1 (0.5)	12 (11.8)	1 (0.5)	2 (2.3)	0 (0.0)
Ethiopia	A3	77 (76.2)	86 (85.1)	12 (11.9)	39 (38.6)	8 (7.9)	2 (2.0)	44 (43.6)	2 (2.0)	9 (8.9)	0 (0.0)
Kenya	A1	7 (4.0)	42 (24.3)	2 (1.2)	3 (1.7)	0 (0)	0 (0)	1 (0.6)	0 (0)	3 (1.7)	0 (0)
Kenya	A2	25 (14.5)	72 (41.4)	6 (3.3)	20 (11.7)	8 (4.8)	3 (1.6)	4 (2.6)	1 (0.6)	4 (2.3)	0 (0.0)
Kenya	A3	55 (31.8)	118 (68.2)	13 (7.5)	53 (30.6)	23 (13.3)	7 (4.0)	10 (5.8)	3 (1.7)	6 (3.5)	0 (0.0)
Mali	A1	13 (17.6)	17 (23.0)	0 (0)	0 (0)	1 (1.4)	0 (0)	1 (1.4)	0 (0)	1 (1.4)	0 (0)
Mali	A2	16 (20.9)	19 (25.7)	0 (0.0)	0 (0.0)	1 (1.4)	0 (0.0)	3 (4.1)	0 (0.0)	2 (2.0)	0 (0.0)

© 2022 Madewell ZJ et al. *JAMA Network Open.*

Mali	A3	18 (24.3)	21 (28.4)	0 (0.0)	0 (0.0)	1 (1.4)	0 (0.0)	5 (6.8)	0 (0.0)	2 (2.7)	0 (0.0)
Mozambique	A1	78 (30.6)	21 (8.2)	0 (0)	0 (0)	0 (0)	0 (0)	2 (0.8)	0 (0)	0 (0)	0 (0)
Mozambique	A2	100 (39.3)	45 (17.7)	2 (0.7)	2 (0.9)	2 (0.6)	1 (0.6)	11 (4.5)	0 (0.2)	2 (0.6)	0 (0.0)
Mozambique	A3	133 (52.2)	81 (31.8)	4 (1.6)	8 (3.1)	7 (2.7)	7 (2.7)	31 (12.2)	2 (0.8)	5 (2.0)	0 (0.0)
Sierra Leone	A1	6 (4.8)	23 (18.5)	0 (0)	6 (4.8)	0 (0)	0 (0)	3 (2.4)	0 (0)	0 (0)	0 (0)
Sierra Leone	A2	25 (20.4)	44 (35.9)	0 (0.0)	8 (6.7)	1 (1.1)	0 (0.0)	6 (5.1)	0 (0.3)	0 (0.3)	0 (0.0)
Sierra Leone	A3	47 (37.9)	69 (55.6)	0 (0.0)	12 (9.7)	4 (3.2)	0 (0.0)	11 (8.9)	1 (0.8)	1 (0.8)	0 (0.0)
South Africa	A1	38 (8.7)	15 (3.4)	3 (0.7)	1 (0.2)	0 (0)	1 (0.2)	141 (32.3)	1 (0.2)	0 (0)	0 (0)
South Africa	A2	48 (11.1)	21 (4.7)	5 (1.2)	2 (0.4)	0 (0.1)	2 (0.4)	147 (33.7)	4 (0.8)	0 (0.0)	0 (0.0)
South Africa	A3	59 (13.5)	27 (6.2)	8 (1.8)	3 (0.7)	1 (0.2)	3 (0.7)	153 (35.1)	6 (1.4)	0 (0.0)	0 (0.0)
Infant/child											
Bangladesh	A1	0 (0)	0 (0)	0 (0)	1 (25.0)	0 (0)	0 (0)	0 (0)	0 (0)	0 (0)	0 (0)
Bangladesh	A2	0 (0.0)	0 (0.0)	0 (0.0)	2 (37.5)	0 (12.5)	0 (0.0)	0 (0.0)	0 (0.0)	0 (0.0)	0 (0.0)
Bangladesh	A3	0 (0.0)	0 (0.0)	0 (0.0)	2 (50.0)	1 (25.0)	0 (0.0)	0 (0.0)	0 (0.0)	0 (0.0)	0 (0.0)
Ethiopia	A2	1 (3.8)	4 (16.1)	0 (0.0)	7 (25.2)	5 (20.4)	0 (0.0)	2 (6.1)	4 (15.9)	0 (0.6)	2 (9.2)
Ethiopia	A3	3 (11.5)	18 (69.2)	0 (0.0)	25 (96.2)	22 (84.6)	0 (0.0)	8 (30.8)	18 (69.2)	1 (3.8)	10 (38.5)
Kenya	A1	1 (0.4)	32 (13.4)	0 (0)	8 (3.4)	2 (0.8)	2 (0.8)	0 (0)	3 (1.3)	0 (0)	0 (0)
Kenya	A2	6 (2.4)	84 (35.2)	4 (1.5)	48 (20.0)	22 (9.3)	8 (3.3)	3 (1.4)	27 (11.2)	1 (0.4)	2 (1.0)
Kenya	A3	18 (7.6)	169 (71.0)	12 (5.0)	118 (49.6)	62 (26.1)	22 (9.2)	11 (4.6)	73 (30.7)	4 (1.7)	9 (3.8)
Mali	A1	0 (0)	4 (7.7)	0 (0)	2 (3.8)	4 (7.7)	2 (3.8)	1 (1.9)	4 (7.7)	0 (0)	3 (5.8)
Mali	A2	0 (0.0)	6 (11.2)	0 (0.0)	3 (5.8)	6 (11.5)	3 (5.8)	1 (2.6)	4 (8.7)	0 (0.0)	3 (5.8)
Mali	A3	0 (0.0)	8 (15.4)	0 (0.0)	4 (7.7)	8 (15.4)	4 (7.7)	2 (3.8)	5 (9.6)	0 (0.0)	3 (5.8)
Mozambique	A1	0 (0)	39 (27.3)	0 (0)	2 (1.4)	1 (0.7)	7 (4.9)	2 (1.4)	3 (2.1)	0 (0)	2 (1.4)
Mozambique	A2	1 (0.8)	56 (39.4)	0 (0.3)	7 (4.8)	7 (4.7)	11 (7.9)	15 (10.4)	9 (6.3)	1 (0.9)	3 (2.0)
Mozambique	A3	3 (2.1)	88 (61.5)	1 (0.7)	21 (14.7)	22 (15.4)	22 (15.4)	41 (28.7)	27 (18.9)	5 (3.5)	6 (4.2)
Sierra Leone	A1	0 (0)	11 (6.2)	0 (0)	6 (3.4)	17 (9.7)	1 (0.6)	1 (0.6)	0 (0)	0 (0)	0 (0)
Sierra Leone	A2	2 (1.1)	38 (21.7)	0 (0.3)	25 (14.2)	50 (28.6)	2 (1.2)	10 (5.5)	15 (8.3)	0 (0.1)	1 (0.3)
Sierra Leone	A3	6 (3.4)	85 (48.3)	1 (0.6)	61 (34.7)	106 (60.2)	4 (2.3)	25 (14.2)	44 (25.0)	1 (0.6)	2 (1.1)
South Africa	A1	1 (0.5)	12 (5.4)	0 (0)	2 (0.9)	13 (5.9)	9 (4.1)	42 (19.0)	1 (0.5)	0 (0)	0 (0)
South Africa	A2	5 (2.1)	18 (7.9)	0 (0.2)	3 (1.5)	26 (11.6)	15 (6.7)	50 (22.7)	5 (2.4)	0 (0.0)	0 (0.0)
South Africa	A3	10 (4.5)	25 (11.3)	1 (0.5)	6 (2.7)	41 (18.6)	23 (10.4)	60 (27.1)	10 (4.5)	0 (0.0)	0 (0.0)

© 2022 Madewell ZJ et al. *JAMA Network Open.*

eTable 6. Number and Proportion of Deaths That Could Have Been Prevented From the Optimal Combinations of Health System Improvement Categories Assuming All Recommendations Given for a Single Death Are Necessary to Prevent That Death by Site and Age Group, December 2016 to December 2021

	**Stillbirth**		**Neonate**		**Infant/child**	
Site	**Categories**	**N (%)**	**Categories**	**N (%)**	**Categories**	**N (%)**
Bangladesh	B	68 (37.2)	B	34 (19.2)	C	1 (25.0)
Bangladesh	AB	86 (47.0)	AB	93 (52.5)	CE	2 (50.0)
Bangladesh	ABC	100 (54.6)	ABC	111 (62.7)	CDE	2 (50.0)
Bangladesh	ABCD	103 (56.3)	ABCD	125 (70.6)	CDEF	2 (50.0)
Bangladesh	ABCDH	106 (57.9)	ABCDH	129 (72.9)	BCEGH	2 (50.0)
Ethiopia	B	9 (4.3)	A	1 (1.0)	--	--
Ethiopia	BC	24 (11.6)	AB	12 (11.9)	AC	1 (3.8)
Ethiopia	ABC	43 (20.8)	ABC	24 (23.8)	ACI	2 (7.7)
Ethiopia	ABCH	54 (26.1)	ABCD	39 (38.6)	ACEF	7 (26.9)
Ethiopia	ABCEH	65 (31.4)	ABCDH	46 (45.5)	ACEFI	13 (50.0)
Kenya	A	30 (18.0)	A	42 (24.3)	A	32 (13.4)
Kenya	AB	67 (40.1)	AB	60 (34.7)	AC	61 (25.6)
Kenya	ABC	83 (49.7)	ABC	79 (45.7)	ACF	91 (38.2)
Kenya	ABCE	99 (59.3)	ABCE	94 (54.3)	ACEF	127 (53.4)
Kenya	ABCEG	112 (67.1)	ABCEH	105 (60.7)	ACEFG	140 (58.8)
Mali	B	36 (43.9)	A	17 (23.0)	A	4 (7.7)
Mali	AB	45 (54.9)	AB	32 (43.2)	AE	10 (19.2)
Mali	ABE	47 (57.3)	ABD	36 (48.6)	ACE	14 (26.9)
Mali	ABEJ	49 (59.8)	ABDJ	38 (51.4)	ACEF	18 (34.6)
Mali	ABDEJ	49 (59.8)	ABDEJ	39 (52.7)	ACEFG	22 (42.3)
Mozambique	B	110 (43.0)	B	78 (30.6)	A	39 (27.3)
Mozambique	AB	125 (48.8)	AB	125 (49.0)	AD	53 (37.1)
Mozambique	ABD	134 (52.3)	ABD	145 (56.9)	ADG	64 (44.8)
Mozambique	ABDJ	138 (53.9)	ABDH	149 (58.4)	ADFG	71 (49.7)
Mozambique	ABCDJ	142 (55.5)	ABDHJ	152 (59.6)	ADEFG	85 (59.4)
Sierra Leone	A	16 (13.3)	A	23 (18.5)	E	17 (9.7)
Sierra Leone	AB	57 (47.5)	AB	61 (49.2)	AE	43 (24.4)
Sierra Leone	ABC	76 (63.3)	ABD	72 (58.1)	ACE	72 (40.9)
Sierra Leone	ABCE	82 (68.3)	ABCD	80 (64.5)	ACEF	105 (59.7)
Sierra Leone	ABCEG	83 (69.2)	ABCDE	84 (67.7)	ACDEF	127 (72.2)
South Africa	B	23 (13.1)	D	141 (32.3)	D	42 (19.0)
South Africa	AB	30 (17.1)	BD	187 (42.9)	DE	62 (28.1)
South Africa	ABG	32 (18.3)	ABD	210 (48.2)	ADE	79 (35.7)
South Africa	ABCG	33 (18.9)	ABDH	217 (49.8)	ADEG	94 (42.5)
South Africa	ABCGH	34 (19.4)	ABDFH	221 (50.7)	ADEFG	104 (47.1)

A: Improved clinical management and quality of care
B: Improved antenatal and obstetric care and management
C: Improved health-seeking behavior

© 2022 Madewell ZJ et al. *JAMA Network Open.*

D: Improved infection prevention and control
E: Improved health education [immunizations, preventing malnutrition, diarrhea, malaria, burns, poisoning, etc.]
F: Improved nutritional support
G: Improved HIV prevention and control
H: Improved family planning
I: Improved use of existing vaccinations
J: Improved transport system

© 2022 Madewell ZJ et al. *JAMA Network Open.*

eTable 7. The Number and Proportion of Deaths for Each of the Top 5 Causes of Death for Each Age Group That Could Have Been Prevented for the Top Combination of 1 to 3 Health System Improvement Categories, Assuming All the Recommendations Given for a Single Death Are Necessary to Prevent That Death and the Death Would Be Prevented When Any Single Cause of Death Was Prevented, December 2016 to December 2021

		**One category**		**Two categories**		**Three categories**	
Cause of death	**Total deaths**	**Health system improvement**	**Deaths prevented N (%)**	**Health system improvement**	**Deaths prevented N (%)**	**Health system improvement**	**Deaths prevented N (%)**
Stillbirth							
Perinatal asphyxia/hypoxia	952	B	250 (26)	AB	400 (42)	ABC	466 (49)
Congenital birth defects	95	B	6 (6)	AB	9 (9)	ABC	11 (12)
Other neonatal disorders	73	B	23 (32)	AB	30 (41)	ABC	34 (47)
Sepsis	63	B	11 (17)	BC	13 (21)	ABC	15 (24)
Congenital infection	49	B	7 (14)	BD	12 (24)	ABD	15 (31)
Neonate							
Preterm birth complications	588	D	117 (20)	BD	177 (30)	ABD	269 (46)
Perinatal asphyxia/hypoxia	487	B	111 (23)	AB	260 (53)	ABD	295 (61)
Sepsis	484	D	127 (26)	AD	189 (39)	ABD	270 (56)
Lower respiratory infections	203	D	88 (43)	BD	101 (50)	ABD	126 (62)
Meningitis/Encephalitis	146	D	56 (38)	AD	67 (46)	ABD	87 (60)
Infant/child							
Lower respiratory infections	373	A	40 (11)	AD	77 (21)	ADE	105 (28)
Sepsis	346	D	37 (11)	AD	78 (23)	ADE	110 (32)
Malnutrition	211	F	10 (5)	AF	33 (16)	AEF	69 (33)
Malaria	180	A	25 (14)	AE	45 (25)	ACE	82 (46)
Anemias	122	E	13 (11)	AE	33 (27)	ACE	53 (43)

A: Improved clinical management and quality of care
B: Improved antenatal and obstetric care and management
C: Improved health-seeking behavior
D: Improved infection prevention and control
E: Improved health education [immunizations, preventing malnutrition, diarrhea, malaria, burns, poisoning, etc.]
F: Improved nutritional support
G: Improved HIV prevention and control
H: Improved family planning
I: Improved use of existing vaccinations
J: Improved transport system

For the top five causes of death across all sites for each age group, we identified the top combinations of one to three health system improvement categories that could have prevented the most deaths. For example, malaria was the fourth most frequently listed cause of death for infants/children across all sites. The top combination of three categories that could have prevented the most malaria deaths among infants/children were improved clinical management, health-seeking behavior, and health education, which could have prevented 47.4% (55/116) of deaths, assuming that for deaths with multiple causes, preventing malaria would prevent the death.

© 2022 Madewell ZJ et al. *JAMA Network Open.*

eTable 8. The Number and Proportion of Deaths for Each of the Top 5 Causes of Death for Each Age Group That Could Have Been Prevented for the Top Combination of 1 to 3 Health System Improvement Categories, Assuming All the Recommendations Given for a Single Death Are Necessary to Prevent That Death, With Deaths Weighted by Giving 50% to the Underlying Cause With the Remaining Weight Distributed Evenly Among the Other Causes, December 2016 to December 2021

		One category		Two categories		Three categories	
Cause of death	**Total deaths**	**Health system improvement**	**Deaths prevented N (%)**	**Health system improvement**	**Deaths prevented N (%)**	**Health system improvement**	**Deaths prevented N (%)**
Stillbirth							
Perinatal asphyxia/hypoxia	889	B	234 (26)	AB	376 (42)	ABC	437 (49)
Congenital birth defects	84	B	4 (5)	BC	6 (7)	BCF	9 (11)
Sepsis	56	B	10 (18)	BD	12 (22)	ABD	13 (23)
Congenital infection	42	B	6 (14)	BD	11 (26)	ABD	14 (33)
Other neonatal disorders	39	B	12 (29)	AB	15 (38)	ABC	17 (44)
Neonate							
Preterm birth complications	410	D	71 (17)	BD	111 (27)	ABD	173 (42)
Perinatal asphyxia/hypoxia	352	B	95 (27)	AB	200 (57)	ABD	219 (62)
Sepsis	210	A	36 (17)	AD	72 (34)	ABD	107 (51)
Congenital birth defects	91	B	6 (7)	AB	11 (12)	ABD	16 (17)
Lower respiratory infections	58	D	14 (25)	AD	19 (33)	ABD	26 (45)
Infant/child							
Lower respiratory infections	148	A	23 (16)	AC	36 (24)	ACE	48 (32)
Malaria	119	A	19 (16)	AC	34 (29)	ACE	63 (53)
Sepsis	114	A	16 (14)	AD	29 (25)	ADE	38 (33)
Malnutrition	92	E	5 (5)	EF	14 (16)	AEF	32 (35)
Diarrheal Diseases	64	A	13 (20)	AE	21 (32)	ACE	31 (49)

A: Improved clinical management and quality of care
B: Improved antenatal and obstetric care and management
C: Improved health-seeking behavior
D: Improved infection prevention and control
E: Improved health education [immunizations, preventing malnutrition, diarrhea, malaria, burns, poisoning, etc.]
F: Improved nutritional support
G: Improved HIV prevention and control
H: Improved family planning
I: Improved use of existing vaccinations
J: Improved transport system

When weighting deaths using the 50% underlying or causal chain methods, malaria was the second leading cause of death among infants/children. In this example, improved clinical management, health-seeking behavior, and health education were optimal to prevent the most malaria deaths and could have prevented 55.8% (48/86) for the 50% underlying (eTable 2) and 55.2% (48/87) for the causal chain (eTable 3) methods. The estimated number of deaths that would have been prevented were similar between these alternative weighting methods for all causes of death.

© 2022 Madewell ZJ et al. *JAMA Network Open.*

eTable 9. The Number and Proportion of Deaths for Each of the Top 5 Causes of Death for Each Age Group That Could Have Been Prevented for the Top Combination of 1 to 3 Health System Improvement Categories, Assuming All the Recommendations Given for a Single Death Are Necessary to Prevent That Death, With Deaths Weighted Using a Causal Chain Approach With Greater Weight Given to Underlying, Comorbid, and Immediate Causes of Death, Respectively, December 2016 to December 2021

		**One category**		**Two categories**		**Three categories**	
Cause of death	**Total deaths**	**Health system improvement**	**Deaths prevented N (proportion)**	**Health system improvement**	**Deaths prevented N (proportion)**	**Health system improvement**	**Deaths prevented N (proportion)**
Stillbirth							
Perinatal asphyxia/hypoxia	890	B	234 (26)	AB	377 (42)	ABC	438 (49)
Congenital birth defects	86	B	5 (6)	BC	7 (8)	BCF	10 (11)
Sepsis	56	B	10 (17)	BD	12 (21)	BCD	13 (23)
Congenital infection	43	B	6 (15)	BD	11 (26)	ABD	14 (33)
Other neonatal disorders	37	B	11 (30)	AB	14 (39)	ABC	17 (45)
Neonate							
Preterm birth complications	410	D	71 (17)	BD	111 (27)	ABD	171 (42)
Perinatal asphyxia/hypoxia	365	B	97 (27)	AB	207 (57)	ABD	226 (62)
Sepsis	188	A	35 (18)	AD	63 (34)	ABD	94 (50)
Congenital birth defects	95	B	7 (7)	AB	12 (13)	ABD	16 (17)
Lower respiratory infections	59	D	16 (26)	AD	20 (34)	ABD	27 (46)
Infant/child							
Lower respiratory infections	143	A	23 (16)	AC	35 (25)	ACD	47 (33)
Malaria	122	A	19 (16)	AC	34 (28)	ACE	64 (53)
Malnutrition	98	F	5 (5)	AF	15 (15)	AEF	33 (34)
Sepsis	97	A	14 (14)	AD	25 (26)	ADE	33 (34)
Diarrheal Diseases	67	A	13 (19)	AE	21 (31)	ACE	32 (47)

A: Improved clinical management and quality of care
B: Improved antenatal and obstetric care and management
C: Improved health-seeking behavior
D: Improved infection prevention and control
E: Improved health education [immunizations, preventing malnutrition, diarrhea, malaria, burns, poisoning, etc.]
F: Improved nutritional support
G: Improved HIV prevention and control
H: Improved family planning
I: Improved use of existing vaccinations
J: Improved transport system

© 2022 Madewell ZJ et al. *JAMA Network Open.*

eMethods. Statistical Analysis

For the three most frequent health system improvement categories for each age group, we identified the 20 most frequent causes of death anywhere in the causal chain for which those categories were recommended to prevent the death. Additionally, for neonates and infants/children we evaluated the proportion of the five most frequent causes of death anywhere in the causal pathway that were deemed preventable by the DeCoDe panel. We also calculated the number of deaths that could have been prevented for the optimal combinations of 1-10 health system improvement categories by month from 2017-2021.

We then explored which combinations of health system improvement categories could have prevented the most deaths for each of the top five causes of death for each age group. For this analysis, we identified the optimal combinations of categories by generating every combination of one to three categories and calculating how many deaths for each of the top five causes could have been prevented for each combination, assuming all categories recommended for a single death are necessary to prevent that death. This analysis also assumed that preventing any single unique cause of death for deaths with multiple causes would prevent the death. In this method every unique cause of death had a weight of one, so deaths with multiple causes had weight greater than one. We also conducted a sensitivity analysis by assigning weights to multiple causes of death. We first used a strategy proposed by Piffaretti[1] that assigns 50% of the weight to the underlying cause with the remaining weight distributed evenly among the other causes. Let w_U be the weight for the underlying cause, w_i be the weight for cause i, and m be the number of causes, then: $w_U = 0.5$; $w_i = \frac{1-w_U}{m-1}$. We also used a novel approach by weighting multiple causes in the causal chain with greater weight given to underlying, comorbid, and immediate causes of death, in that order; the rationale behind this method is that each cause of death in the sequence may predispose the individual to subsequent causes. Let c_i be the position along the causal chain, then: $w_i = \frac{(m+1-c_i)}{\sum_{j:c_j \leq m} c_j}$. The sum of weights for the sensitivity analyses for each death is one. Therefore, these sensitivity analyses assume that for deaths with multiple causes, health system improvement categories would prevent a fraction of deaths depending on the cause's position in the causal chain. An example with four causes of death is shown below:

Example of weights applied to causes of death for a death with four causes.

Cause of death	Type	Equal	50% Underlying	Causal chain
HIV	Underlying	1	0.50	0.40
Malaria	Antecedent 2	1	0.17	0.30
Anemia	Antecedent 1	1	0.17	0.20
Tuberculosis	Immediate	1	0.17	0.10

Reference

1. Piffaretti C, Moreno-Betancur M, Lamarche-Vadel A, Rey G. Quantifying cause-related mortality by weighting multiple causes of death. *Bull World Health Organ*. 2016;94(12):870-879. doi:10.2471/BLT.16.172189

© 2022 Madewell ZJ et al. *JAMA Network Open.*
