# Supplementary material for: Prioritizing Health Care Strategies to Reduce Childhood Mortality
Source: JAMA Netw Open. 2022 Oct 21;5(10):e2237689. doi: 10.1001/jamanetworkopen.2022.37689 (PMC9587481; doi:10.1001/jamanetworkopen.2022.37689)
Supplement: Supplement 2. — Nonauthor Collaborators [file jamanetwopen-e2237689-s002.pdf]

Supplemental Online Content: Nonauthor Collaborators
*First name, last name, and suffix (if applicable) are required and will appear in PubMed.

*Group Name(s): Child Health and Mortality Prevention Surveillance Network							
***First Name and Middle Initial(s)**	***Last Name**	***Suffix (eg, Jr, III)**	Academic Degrees	Institution	Location (city, state/province, country)	Role or Contribution, eg, chair, principal investigator	Group (if more than 1 Group listed in the byline) and/or Subgroup (eg, Steering Committee)
Ferdousi	Begum		MD	Bangabandhu Sheikh Mujib Medical Univ	Bangladesh	Data collection	
Mohammad Mosiur	Rahman		MD	Bangabandhu Sheikh Mujib Medical Univ	Bangladesh	Data collection	
Mohammed	Kamal		PhD	Bangabandhu Sheikh Mujib Medical Univ	Bangladesh	Data collection	
A.S.M. Nawshad Uddi	Ahmed		FCPS	Bangladesh Institute of Child Health at D	Bangladesh	Data collection	
Mahbubul	Hoque		FCPS	Bangladesh Institute of Child Health at D	Bangladesh	Data collection	
Kyu Han	Lee		PhD	Department of Epidemiology, Johns Hop	Bangladesh	Data collection	
Saria	Tasnim		FCPS	Dhaka Community Medical College and H	Bangladesh	Data collection	
Mahbubur	Rahman		MPH	Institute of Epidemiology, Disease Contro	Bangladesh	Data collection	
Tahmina	Shirin		PhD	Institute of Epidemiology, Disease Contro	Bangladesh	Data collection	
Iqbal Ansary	Khan		MPH	Institute of Epidemiology, Disease Contro	Bangladesh	Data collection	
Meerjady Sabrina	Flora		PhD	Institute of Epidemiology, Disease Contro	Bangladesh	Data collection	
Dilruba	Ahmed		PhD	International Centre for Diarrhoeal Disea	Bangladesh	Data collection	
K.	Zaman		PhD	International Centre for Diarrhoeal Disea	Bangladesh	Data collection	
Kazi Munisul	Islam		MPH	International Centre for Diarrhoeal Disea	Bangladesh	Data collection	
Mohammed Sabbir	Ahmed		MPH	International Centre for Diarrhoeal Disea	Bangladesh	Data collection	
Mohammed Zahid	Hossain		PhD	International Centre for Diarrhoeal Disea	Bangladesh	Data collection	
Mustafizur	Rahman		PhD	International Centre for Diarrhoeal Disea	Bangladesh	Data collection	
Shahana	Parveen			International Centre for Diarrhoeal Disea	Bangladesh	Data collection	
Sanwarul	Bari		MD	Maternal and Child Health Division, Inter	Bangladesh	Data collection	
Farida	Arjuman		FCPS	National Institute of Cancer Research and	Bangladesh	Data collection	
Farzana	Islam		PhD	PEI, International Center for Diarrhoeal D	Bangladesh	Data collection	
Ferdousi	Islam		FCPS	Popular Medical College and Hospital in I	Bangladesh	Data collection	
Joseph O.	Oundo			1) London School of Hygiene & Tropical M	Ethiopia	Data collection	
Gunturu	Revathi		MD	Aga Khan University Hospital, Nairobi, Ke	Kenya	Data collection	
Emily	Rogena		MMED	Jomo Kenyatta University of Agriculture a	Kenya	Data collection	
Magdalene	Kuria		MMED	Kisumu County Department of Health	Kenya	Data collection	
Paul K.	Mitei		MMED	Kisumu County Department of Health	Kenya	Data collection	
Florence	Murila		MMED	University of Nairobi	Kenya	Data collection	
Jennifer	Verani		MD	CDC Atlanta	U.S.A.	Data collection	

Supplemental Online Content: Nonauthor Collaborators
*First name, last name, and suffix (if applicable) are required and will appear in PubMed.

*First Name and Middle Initial(s)	*Last Name	*Suffix (eg, Jr, III)	Academic Degrees	Institution	Location (city, state/province, country)	Role or Contribution, eg, chair, principal investigator	Group (if more than 1 Group listed in the byline) and/or Subgroup (eg, Steering Committee)
Doh	Sanogo			Centre pour le Développement des Vacci	Mali	Data collection	
Nana	Kourouma			Centre pour le Développement des Vacci	Mali	Data collection	
Seydou	Sissoko			Centre pour le Développement des Vacci	Mali	Data collection	
Uma U.	Onwuchekwu			Centre pour le Développement des Vacci	Mali	Data collection	
Diakaridia	Sidibe			Centre pour le Développement des Vacci	Mali	Data collection	
Tatiana	Keita		MD	Clinique Pasteur, Bamako, Mali	Mali	Data collection	
Diakaridia	Kone			CSRef Commune I, Bamako, Mali	Mali	Data collection	
Sharon M.	Tennant		PhD	University of Maryland School of Medicir	U.S.A.	Data collection	
Carol L.	Greene		MD	University of Maryland School of Medicir	U.S.A.	Data collection	
J. Kristie	Johnson		PhD	University of Maryland School of Medicir	U.S.A.	Data collection	
Rima	Koka		MD	University of Maryland School of Medicir	U.S.A.	Data collection	
Karen D.	Fairchild		MD	University of Virginia	U.S.A.	Data collection	
Sandra	Lako		MPH	Aberdeen Women's Centre in Freetown	Sierra Leone	Data collection	
Erick	Kaluma		MPH	Crown Agents	Sierra Leone	Data collection	
Samuel	Pratt		MPH	FOCUS 1000	Sierra Leone	Data collection	
Baindu	Kosia		MPH	Johns Hopkins Program for International	Sierra Leone	Data collection	
Ruby	Fayorsey		MPH	ICAP at Columbia University and Harlem	Sierra Leone	Data collection	
Rebecca Alkis	Ramirez		MPH	Center for Global Health, Centers for Dis	U.S.A.	Data collection	
Rebecca P.	Philipsborn		MD	Department of Pediatrics, Emory Univers	U.S.A.	Data collection	
Mischka	Garel		MPH	Emory Global Health Institute, Emory Un	U.S.A.	Data collection	
Navit T.	Salzberg		MPH	Emory Global Health Institute, Emory Un	U.S.A.	Data collection	
Jeffrey P.	Koplan		MD	Emory Global Health Institute, Emory Un	U.S.A.	Data collection	
Robert F.	Breiman		MD	Emory Global Health Institute, Emory Un	U.S.A.	Data collection	
Jana M.	Ritter		DVM	Infectious Diseases Pathology Branch, Di	U.S.A.	Data collection	
Joy	Gary		DVM	Infectious Diseases Pathology Branch, Di	U.S.A.	Data collection	
Sherif R. (deceased)	Zaki		MD	Infectious Diseases Pathology Branch, Di	U.S.A.	Data collection	
Jennifer R.	Verani		MD	National Center for Immunization and Re	U.S.A.	Data collection	
Jacob	Witherbee			National Center for Immunization and Re	U.S.A.	Data collection	
Lucy	Liu		MBA	Public Health Informatics Institute at the	U.S.A.	Data collection	
Shailesh	Nair		MPH	Public Health Informatics Institute, The T	U.S.A.	Data collection	
Jonas M.	Winchell		PhD	Respiratory Diseases Branch, Division of	U.S.A.	Data collection	

Supplemental Online Content: Nonauthor Collaborators
*First name, last name, and suffix (if applicable) are required and will appear in PubMed.

*First Name and Middle Initial(s)	*Last Name	*Suffix (eg, Jr, III)	Academic Degrees	Institution	Location (city, state/province, country)	Role or Contribution, eg, chair, principal investigator	Group (if more than 1 Group listed in the byline) and/or Subgroup (eg, Steering Committee)
Joseph O.	Oundo		PhD	1) London School of Hygiene & Tropical M	Ethiopia	Decode Panel	
Fikremelekot	Temesgen		MD	Addis Ababa University	Ethiopia	Decode Panel	
Melisachew Mulatu	Yeshi		MD	Ayder Specialized Comprehensive Hospit	Ethiopia	Decode Panel	
Addisu	Alemu		MD	College of Health and Medical Sciences a	Ethiopia	Decode Panel	
Alexander M.	Ibrahim		MD	College of Health and Medical Sciences a	Ethiopia	Decode Panel	
Tadesse	Gure		MD	College of Health and Medical Sciences a	Ethiopia	Decode Panel	
Stian	Orlien		PhD	London School of Hygiene & Tropical Me	Ethiopia	Decode Panel	
Solomon	Ali		PhD	National Data Management Centre at the	Ethiopia	Decode Panel	
Yunus	Edrid		MD	National Data Management Centre at the	Ethiopia	Decode Panel	
Mahlet Abayneh	Gizaw		MD	St. Paul’s Hospital Millennium Medical Co	Ethiopia	Decode Panel	
Fentabil	Getnet		PhD	Ethiopian Public Health Institute	Ethiopia	Decode Panel	
Surafel	Fentaw		Mphil	Ethiopian Public Health Institute	Ethiopia	Decode Panel	
Celso	Monjane		MD	Instituto Nacional de Saúde (INS) in Map	Mozambique	Data collection	
Sheila	Nhachungue		MD	Instituto Nacional de Saúde (INS) in Map	Mozambique	Data collection	
Clara	Menendez		PhD	1) ISGlobal; 2) Centro de Investigação em	Mozambique	Data collection	
Justina	Bramugy		MD	Centro de Investigação em Saúde de Mar	Mozambique	Data collection	
Milton	Kindcardett		MD	Centro de Investigação em Saúde de Mar	Mozambique	Data collection	
Taciltа	Nhampossa		PhD	Centro de Investigação em Saúde de Mar	Mozambique	Data collection	
Ariel	Nhacolo		MSc	Centro de Investigação em Saúde de Mar	Mozambique	Data collection	
Khátia	Munguambe		PhD	Centro de Investigacao em Saude de Mar	Mozambique	Data collection	
Pio	Vitorino		MSc	Centro de Investigacao en Saude de Man	Mozambique	Data collection	
Carla	Carrilho		Phd	Department of Pathology, Maputo Centr	Mozambique	Data collection	
Fabiola	Fernandes		PhD	Department of Pathology, Maputo Centr	Mozambique	Data collection	
Sibone	Mocumbi		PhD	Eduarado Mondlane University and Map	Mozambique	Data collection	
Maria	Maixenchs		PhD	IS Global Hospital Clinic--Universitat de B	Mozambique	Data collection	
Juan Carlos	Hurtado		MD	ISGlobal - Barcelona Spain	Mozambique	Data collection	
Jaume	Ordi		PhD	ISGlobal - Hospital Clínic, Unversitat de B	Mozambique	Data collection	
Marta	Valente		MD	ISGlobal Hospital Clinic at Universitat de	Mozambique	Data collection	
Natalia	Rakislova		PhD	ISGlobal; Department of Pathology, Hosp	Mozambique	Data collection	
Dercio	Chitungo		MD	Quelimane Central Hospital	Mozambique	Data collection	
Zara	Manhique		MD	Quelimane Central Hospital	Mozambique	Data collection	

Supplemental Online Content: Nonauthor Collaborators
*First name, last name, and suffix (if applicable) are required and will appear in PubMed.

***First Name and Middle Initial(s)**	***Last Name**	***Suffix (eg, Jr, III)**	Academic Degrees	Institution	Location (city, state/province, country)	Role or Contribution, eg, chair, principal investigator	Group (if more than 1 Group listed in the byline) and/or Subgroup (eg, Steering Committee)
Yasmin	Adam		MD	1) Department of Obstetrics & Gynaecolo	South Africa	Data collection	
Sanjay G.	Lala		MD	Department of Paediatrics and Perinatal	South Africa	Data collection	
Karen L.	Petersen		MD	Department of Paediatrics, Chris Hani Ba	South Africa	Data collection	
Peter J.	Swart		MD	National Health for Laboratory Service in	South Africa	Data collection	
Martin	Hale		MD	National Health Laboratory Service, Depa	South Africa	Data collection	
Jeannette	Wadula		MD	National Health Laboratory Service, Depa	South Africa	Data collection	
Noluthando	Dludlu		BCMP	South African Council Vaccines and Infec	South Africa	Data collection	
Fatima	Solomon		MD	South African Medical Research Council V	South Africa	Data collection	
Richard	Chawana		PhD	South African Medical Research Council V	South Africa	Data collection	
Hennie	Lombaard		MD	University of Witwatersrand, Johannesbu	South Africa	Data collection	
Gillian	Sorour		MD	Wits Health Consortium	South Africa	Data collection	
Constance	Ntuli		Auxillary nu	South African Council Vaccines and Infec	South Africa	Data collection	
